# Non-alcoholic fatty liver disease promotes liver metastasis of colorectal cancer via fatty acid synthase dependent EGFR palmitoylation

**DOI:** 10.1038/s41420-023-01770-x

**Published:** 2024-01-23

**Authors:** Chi Zhang, Yue Zhang, Yan Dong, Ruiyang Zi, Yijie Wang, Yanrong Chen, Chengxiang Liu, Junyi Wang, Xuesong Wang, Jianjun Li, Houjie Liang, Juanjuan Ou

**Affiliations:** 1 Department of Oncology and Southwest Cancer Centre, Southwest Hospital, Third Military Medical University (Army Medical University), 400038 Chongqing, China; 2 Jinfeng Laboratory, 401329 Chongqing, China

**Keywords:** Metastasis, Gastrointestinal cancer

## Abstract

Liver metastasis is the major reason for most of colorectal cancer (CRC) related deaths. Accumulating evidence indicates that CRC patients with non-alcoholic fatty liver disease (NAFLD) are at a greater risk of developing liver metastasis. With the growing prevalence of NAFLD, a better understanding of the molecular mechanism in NAFLD-driven CRC liver metastasis is needed. In this study, we demonstrated that NAFLD facilitated CRC liver metastasis as a metabolic disorder and promoted the stemness of metastatic CRC cells for their colonization and outgrowth in hepatic niches. Metabolically, the lipid-rich microenvironment in NAFLD activated de novo palmitate biosynthesis in metastatic CRC cells via upregulating fatty acid synthase (FASN). Moreover, increased intracellular palmitate bioavailability promoted EGFR palmitoylation to enhance its protein stability and plasma membrane localization. Furthermore, we demonstrated that the FDA-approved FASN inhibitor orlistat could reduce NAFLD-activated endogenous palmitate production, thus inhibiting palmitoylation of EGFR to suppress CRC cell stemness and restrict liver metastasis in synergy with conventional chemotherapy. These findings reveal that the NAFLD metabolic microenvironment boosts endogenous palmitate biosynthesis in metastatic CRC cells and promotes cell stemness via EGFR palmitoylation, and FASN inhibitor orlistat could be a candidate adjuvant drug to suppress liver metastasis in CRC patients with NAFLD.

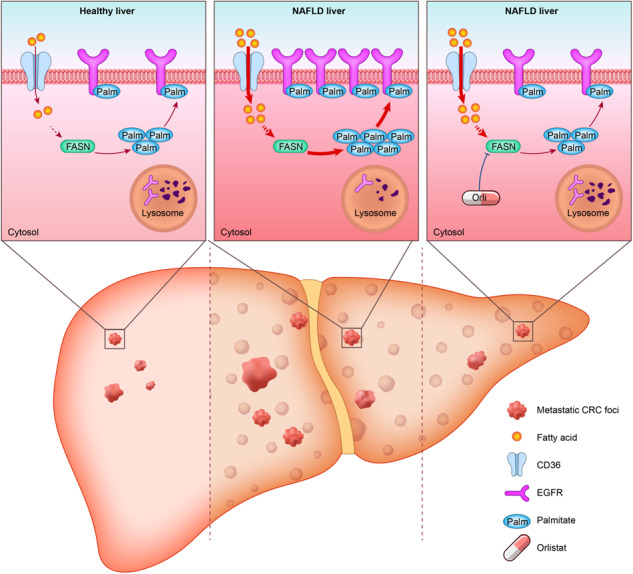

## Introduction

Colorectal cancer (CRC) is the second most common cause of cancer-related mortality worldwide, with more than 935,000 deaths in 2020 [1]. Distant metastasis is the leading cause of CRC-related death, and the liver is the most common organ for CRC metastasis. Despite inspiring progresses have been made in early-diagnostic and therapeutic modalities, the 5-year overall survival rate of CRC patients with unresectable liver metastasis remains only 2.2% [2]. Non-alcoholic fatty liver disease (NAFLD) is the most common liver disease worldwide, with an overall global prevalence of 30%, and its incidence is projected to increase by 56% over the next decade [3]. Recently, a growing body of evidence suggests that NAFLD has become an emerging risk factor for CRC [4, 5]. Therefore, the increasing incidence of NAFLD indicates that the population of patients with both NAFLD and CRC is also rising. Recent clinical observations have raised concerns that CRC patients with NAFLD are at a higher risk of developing liver metastasis and have shorter relapse-free survival after surgery [6–8]. Other laboratory evidence has also suggested that NAFLD could promote liver metastasis of multiple types of cancers in murine models, including CRC [9–11]. However, the underlying mechanisms remain elusive, and targeted therapy for clinical use is currently unavailable.

NAFLD is a systemic metabolic disorder characterized by abnormal lipid accumulation in the liver microenvironment [12]. Although it is well recognized that the tumor metabolic microenvironment could exert a profound influence on multiple biological processes of cancer cells [13], the underlying mechanisms of how the NAFLD metabolic microenvironment facilitates cancer progression remain partially elucidated. A previous study showed that a lipid-rich NAFLD metabolic microenvironment could activate the CEBPβ-Nogo-B-YAP pathway in hepatocellular carcinoma cells [14]. Recently, a study revealed that NAFLD could promote CRC liver metastasis (CRLM) by promoting IL-1β and VEGF production from tumor-associated macrophages (TAMs) educated by the lipid-rich hepatic microenvironment [10]. In our previous report, we found that primary CRC tissues displayed an increased lipid content, which was similar to the NAFLD liver microenvironment, and metabolic communications between the lipid-rich tumor microenvironment and CRC cells promoted their progression [15, 16]. Nevertheless, metabolic crosstalk between the NAFLD hepatic microenvironment and liver metastatic CRC cells is poorly understood.

Reprogrammed lipid metabolism is one of the most remarkable metabolic features of cancer cells [17, 18]. Lipids are utilized in cancer cells as essential signaling molecules and primary nutrient sources for energy generation and macromolecular biomass synthesis [19]. Recently, lipids have been recognized as pivotal substrates for post-translational modifications (PTM), which could directly bind and modify numerous important proteins and play a decisive role in multiple aspects of cellular physiology [20]. Palmitoylation, which is defined as a covalent thioester bond established between 16-carbon palmitate and cysteine residues of proteins, is one of the most well-studied and the only known reversible post-translational lipid modification so far [21]. Palmitoylation is currently found to contribute to tumor initiation and progression in a variety of ways, such as facilitating subcellular trafficking and stability of receptor tyrosine kinase (RTK) c-Met and maintaining plasma membrane (PM) localization of the glucose transporter GLUT1 to sustain aerobic glycolysis [22, 23]. Further studies have revealed that lipid bioavailability is a key regulator of protein palmitoylation [24–26]. In NAFLD, as a consequence of the lipid-rich microenvironment, palmitoylation levels of particular proteins in hepatocytes, such as the fatty acid transporter protein CD36, were found to increase and contributed substantially to disease exacerbation [27, 28]. But whether NAFLD metabolic microenvironment promotes liver metastasis of CRC in a palmitoylation-dependent manner is still far from certain.

The epidermal growth factor receptor (EGFR), a RTK, is positively associated with CRLM and poor survival [29]. Clinically available EGFR-targeting therapies are more beneficial for cancer patients with EGFR mutations; whereas, for most CRC patients with wild-type EGFR, these treatment modalities are blunted [30]. For wild-type EGFR, post-translational modifications (PTM) are especially important for stability, subcellular distribution, and signal transduction [31]. Recent studies have suggested that targeting PTMs of EGFR is a promising strategy for treating tumors harboring wild-type EGFR [32], among which palmitoylation of EGFR has attracted considerable attention due to its particular influence on membrane proteins. But whether the palmitoylation status of EGFR changes and contributes to liver metastasis of CRC remains unclear. In the present study, we demonstrated that the NAFLD metabolic microenvironment promoted liver metastasis of CRC via EGFR palmitoylation. Metabolically, the lipid-rich microenvironment in NAFLD activated de novo palmitate biosynthesis in hepatic metastatic CRC cells by upregulating fatty acid synthase (FASN) expression and boosting endogenous palmitate production to promote EGFR palmitoylation for its PM localization and evasion from lysosomal degradation. Furthermore, we identified that the FDA-approved FASN inhibitor orlistat could interrupt NAFLD-induced lipid metabolic reprogramming, thus inhibiting EGFR palmitoylation and decreasing its stability and PM localization to suppress CRC cell stemness and restrict liver metastasis in synergy with conventional chemotherapy. Collectively, our findings reveal that NAFLD promotes liver metastasis of CRC via enhancing de novo palmitate biosynthesis-induced EGFR palmitoylation and provide a rational therapeutic strategy for liver metastasis in CRC patients with NAFLD.

## Results

### NAFLD facilitates liver metastasis of CRC and promotes the stemness properties of metastatic CRC cells

**Fig. 1:**
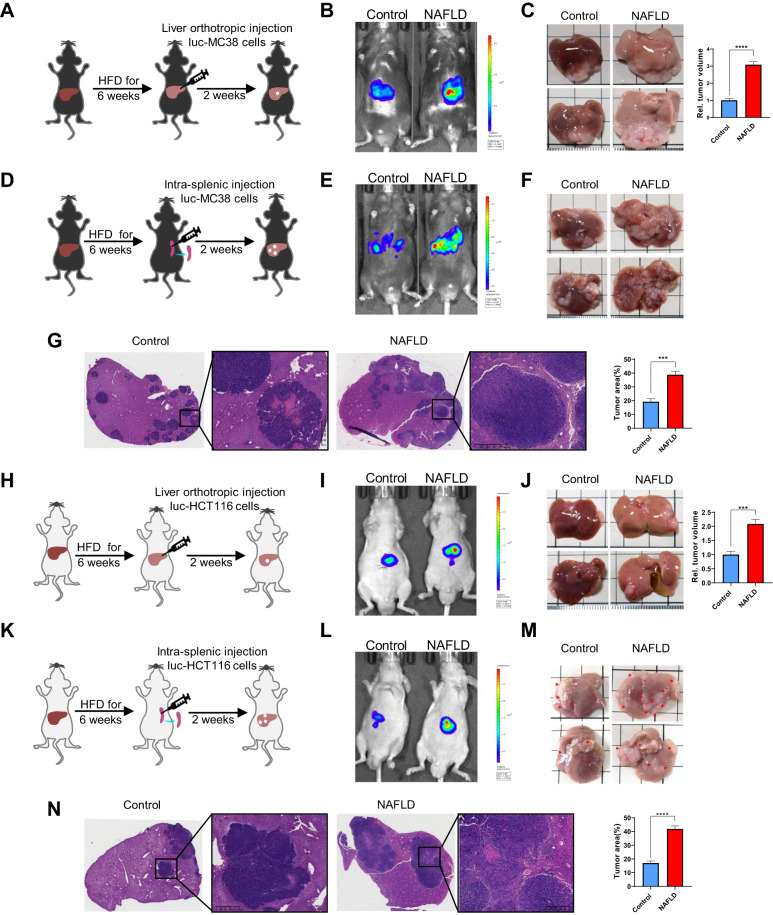
NAFLD promotes liver metastasis of CRC. **A**–**C** Schematics illustrating the experimental liver metastasis model established via liver orthotopic implantation in C57BL/6 mice (**A**). In vivo bioluminescent images of luc-MC38 cells implanted in the liver of control and NAFLD C57BL/6 mice at day 14 after implantation (**B**). At the end of the experiment, liver metastases were taken photos and metastatic lesion volume was assessed (**C**). **D**–**G** Schematics illustrating the experimental liver metastasis model established via splenic implantation in C57BL/6 mice (**D**). In vivo bioluminescent images of luc-MC38 cells injected in the spleen of control and NAFLD C57BL/6 mice at day 14 after implantation (**E**). At the end of the experiment, liver metastases were dissected for taking photos, H&E staining and assessing tumor area (**F**, **G**). **H**–**J** Schematics illustrating the experimental liver metastasis model via liver orthotopic implantation in BALB/c nude mice (**H**). In vivo bioluminescent images of luc-HCT 116 cells implanted in the liver of control and NAFLD nude mice day 14 after implantation (**I**). At the end of the experiment, liver metastases were taken photos and assess metastatic lesion volume (**J**). **K**–**N** Schematics illustrating the experimental liver metastasis model via splenic implantation in BALB/c nude mice (**K**). In vivo bioluminescent images of luc-HCT 116 cells injected in the spleen of control and NAFLD nude mice at day 14 after implantation (**L**). At the end of the experiment, liver metastases were dissected for taking photos, H&E staining and assessing tumor area (**M**, **N**). Data are presented as mean ± SEM. Significance was determined by two-tailed unpaired Student’s *t* test, *p < 0.05, **p < 0.01, ***p < 0.001, ****p < 0.0001. NAFLD non-alcoholic fatty liver disease, HFD high-fat diet, luc-HCT 116 cells Luciferase-labeled HCT 116 cells, luc-MC38 cells Luciferase-labeled MC38 cells.

To determine the effects of NAFLD on CRLM, we continuously fed C57BL/6 mice a high-fat diet (HFD) for 6 weeks to mimic the development of NAFLD in vivo, and validated that NAFLD was successfully established via Oil Red O and BODIPY staining assays (Fig. S1A, B). Then we implanted murine CRC cell MC38 into the liver and spleen respectively to establish two experimental CRLM models in C57BL/6 mice (Fig. 1A, D). After 2 weeks, we examined the effects of NAFLD on CRLM using in vivo bioluminescent imaging, and the results indicated that NAFLD markedly increased the size of CRC metastatic foci in the liver (Fig. 1B, E). Livers were harvested to further evaluate CRC metastasis, compared to mice in the control group, NAFLD mice had a greater number and size of visibly and histologically detectable tumor nodules in the liver (Fig. 1C, F, G). As NAFLD is recognized as a systemic metabolic disorder, we then determined to further investigate whether metabolic dysfunction contributes to CRLM of human CRC cells in immunodeficient NAFLD mouse models. Thus, we fed BALB/c nude mice with HFD for 6 weeks to induce NAFLD in immunodeficient mice models and confirmed that HFD feeding was sufficient to induce NAFLD via histological experiments (Fig. S1C, D). Next, we implanted human CRC cells HCT 116 intrahepatically and intrasplenically to establish two experimental CRLM models (Fig. 1H, K). After 2 weeks, in vivo bioluminescent imaging was conducted, and the results indicated that both hepatic CRC seeding and growth were remarkably increased in the NAFLD group (Fig. 1I, L). The livers were resected to evaluate CRC metastasis, and the results showed that there was a significant increase in both the number and size of metastatic tumor nodules in the livers of the NAFLD group (Fig. 1J, M, N).

**Fig. 2:**
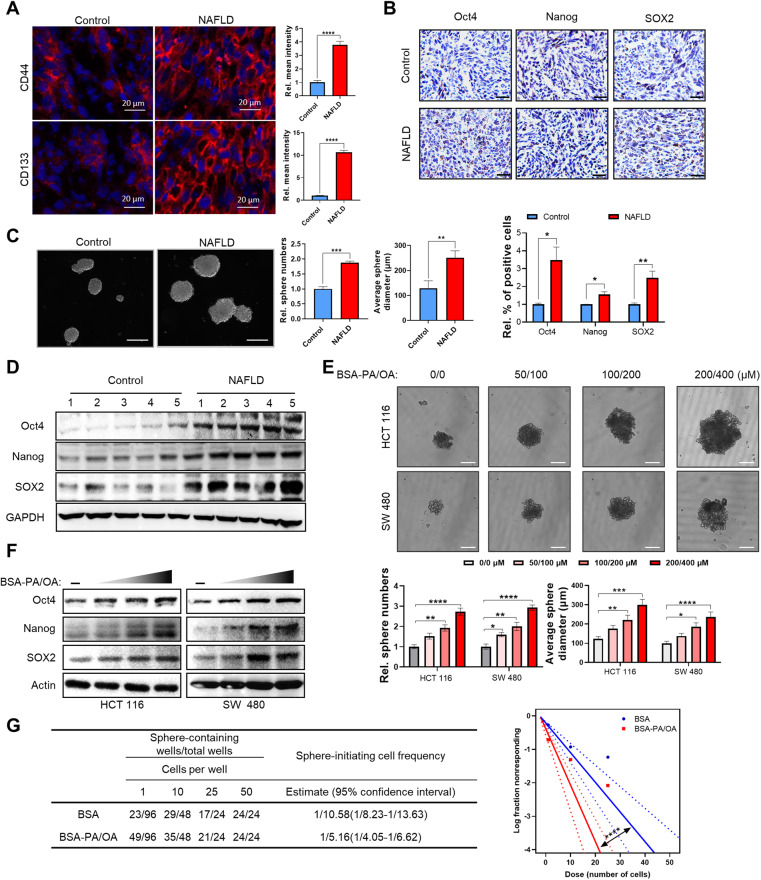
NAFLD promotes the stemness properties of liver metastatic CRC cells. **A** Representative immunofluorescence images for CD44 and CD133 (red) expression of MC38 allografts in control and NAFLD mice with intrahepatic implantation. DAPI (blue) to mark nucleus. Scale bars, 20 μm. Right, quantification of the results (*n* = 5). **B** Representative IHC staining images for Oct4, Nanog and SOX2 of MC38 allografts in control and NAFLD mice with intrahepatic implantation. Scale bars, 50 μm. Below, quantification of the results (*n* = 5). **C** Sphere formation assay on tumor cells isolated from HCT 116 xenografts in control and NAFLD mice. Representative photos of HCT 116 sphere in the second passage were taken on day 10 after cells were seeded (scale bars, 200 μm), and sphere numbers and diameters were determined and plotted (*n* = 3). **D** The protein level of CSC markers Oct4, Nanog and SOX2 of HCT 116 xenografts in control and NAFLD mice by western blot assay (*n* = 5). **E** Sphere formation assay on HCT 116 and SW 480 cells treated with BSA-PA/OA at indicated concentrations (PA 0 µM, OA 0 µM; PA 50 µM, OA 100 µM; PA 100 µM, OA 200 µM; PA 200 µM, OA 400 µM, respectively). Representative photos of sphere in the second passage were taken on day 10 after cells were seeded (scale bars, 100 μm), and sphere numbers and diameters were determined and plotted (*n* = 3). **F** Protein level of CSC markers of HCT 116 and SW 480 cells treated with BSA-PA/OA at different concentrations (PA 0 µM, OA 0 µM; PA 50 µM, OA 100 µM; PA 100 µM, OA 200 µM; PA 200 µM, OA 400 µM, respectively) detected by western blot assay. **G** In vitro limiting dilution analysis for frequency of CSCs in HCT 116 cells treated with BSA or BSA-PA/OA, calculated with Extreme Limiting Dilution Analysis software (http://bioinf.wehi.edu.au/software/elda/). Data are presented as mean ± SEM. Significance was determined by two-tailed unpaired Student’s *t* test (**A**–**C**), one-way ANOVA (**E**) and Pairwise test (**G**), *p < 0.05, **p < 0.01, ***p < 0.001, ****p < 0.0001. NAFLD non-alcoholic fatty liver disease, BSA bovine serum albumin, PA palmitic acid, OA oleic acid.

Cancer stem cells represent an important source of development and progression of CRC, and it is generally acknowledged that cell stemness contributes substantially to the colonization and outgrowth of cancer cells in metastatic niches and disease relapse after resection of the original metastatic lesions [33, 34]. Hence, to elucidate the mechanism underlying CRLM in NAFLD, we evaluated the stemness of liver metastatic CRC cells. We firstly detected the expression of the CRC stemness markers CD44 and CD133 by immunofluorescence (IF) assay and Oct4, Nanog, and SOX2 by immunohistochemistry (IHC) assay in metastatic CRC liver tissues. The results indicated that there was an increased expression of these stemness markers at the protein level in metastatic CRC tissues from the NFALD group (Fig. 2A, B). To further investigate whether NAFLD promoted metastatic CRC cell stemness, we evaluated the sphere formation capacity of primary cultured cells from metastatic CRC xenografts in BALB/c nude mice. The results showed that primary cultured cells from NAFLD liver xenografts formed more and larger spheres (Fig. 2C, Fig. S2A). Western blot assay results also suggested that stemness markers in NAFLD liver xenografts increased at the protein level (Fig. 2D). To better understand the mechanistic contributions of the NAFLD metabolic microenvironment to metastatic CRC stemness, we modeled the lipid-rich NAFLD hepatic metabolic microenvironment in vitro by exposing cultured CRC cells to palmitic acid/oleic acid (PA/OA) treatment as previously reported [35]. The in vitro sphere formation assay results were consistent with in vivo data (Fig. 2E, Fig. S2B). Western blotting results suggested that cell stemness markers were increased in a dose-dependent manner after PA/OA treatment (Fig. 2F). Subsequent in vitro limiting dilution spheroid formation assays also revealed that the stemness of CRCs was increased in PA/OA treated cells (Fig. 2G).

Collectively, these results indicated that the NAFLD metabolic environment promoted CRLM and the stemness of hepatic metastatic CRC cells.

### NAFLD promotes the stemness properties of liver metastatic CRC cells in a palmitoylation-dependent manner

**Fig. 3:**
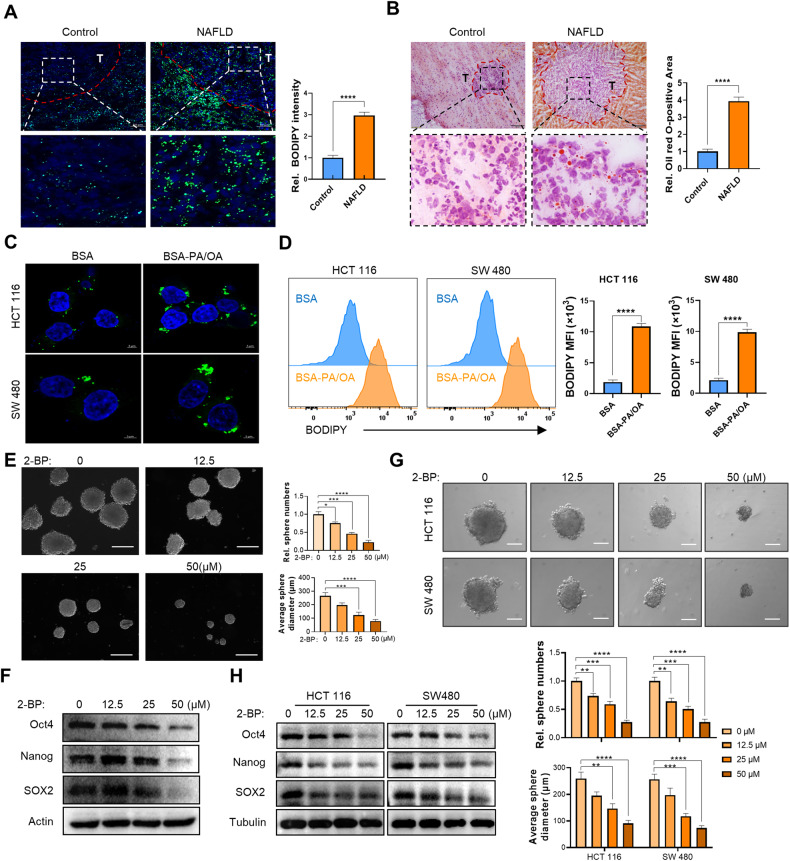
NAFLD promotes metastatic CRC cell stemness in a palmitoylation-dependent manner. **A** Representative fluorescence imaging of BODIPY 493/503 staining (green), and corresponding quantification data in MC38 allografts in control and NAFLD mice (*n* = 5). Nuclei were stained with DAPI (blue). scale bars, 50 μm. **B** Representative imaging of Oil Red O staining, and corresponding quantification data in MC38 allografts in control and NAFLD mice (*n* = 5). scale bars, 100 μm. **C** Representative fluorescence imaging of BODIPY 493/503 staining (green) in HCT 116 and SW 480 cells treated with BSA or BSA-PA/OA (PA 100 μM, OA 200 μM) for 36 h. Nuclei were stained with DAPI (blue). scale bars, 5 μm. **D** Representative quantitative plots of the histogram (left) and MFI (right) of BODIPY 493/503 staining in HCT 116 and SW 480 cells treated with BSA or BSA-PA/OA (PA 100 μM, OA 200 μM) for 36 h. **E** Sphere formation assay on tumor cells isolated from HCT 116 xenografts in NAFLD mice and exposed to indicated concentrations of 2-BP. Representative images of sphere in second passage were taken on day 10 after cells were seeded (scale bars, 200 μm), and sphere numbers and diameters were determined and plotted (*n* = 3). **F** The protein level of CSC markers Oct4, Nanog and SOX2 of indicated concentrations of 2-BP treated tumor cells isolated from HCT 116 xenografts in NAFLD mice by western blot assay. **G** Sphere formation assay in HCT 116 and SW480 cells treated with BSA-PA/OA (PA 100 μM, OA 200 μM) and indicated concentrations of 2-BP. Representative images of sphere in second passage were taken on day 10 after cells were seeded (scale bars, 100 μm), and sphere numbers and diameters were determined and plotted (*n* = 3). **H** The protein level of CSC markers Oct4, Nanog and SOX2 of in HCT 116 and SW 480 cells treated with BSA-PA/OA (PA 100 μM, OA 200 μM) and 2-BP at indicated doses for 24 h by western blot assay. Data are presented as mean ± SEM. Significance was determined by two-tailed unpaired Student’s *t* test (**A**, **B**, **D**) and one-way ANOVA (**E**, **G**), *p < 0.05, **p < 0.01, ***p < 0.001, ****p < 0.0001. NAFLD non-alcoholic fatty liver disease, BSA bovine serum albumin, PA palmitic acid, OA oleic acid, MFI mean fluorescence intensity, 2-BP 2-bromopalmitate.

As is indicated that metabolic reprogramming contributes to CRLM [36] and it has recently been recognized that the tumor metabolic microenvironment has a comprehensive influence on cancer cell metabolism [13], we next sought to investigate whether there is a potential link between the NAFLD metabolic environment and intrinsic metabolism in liver metastatic CRC cells. As is shown in Fig. 3A, B, there was significantly more lipid content in metastatic CRC liver tissues in NAFLD mice. In vitro experiments showed that PA/OA treatment promoted intracellular lipid accumulation in CRC cells (Fig. 3C, D).

Recently, lipids have been acknowledged as essential substrates for the specific binding and modification of various proteins [21], and palmitoylation is one of the most well-established and only known reversible post-translational lipid modification up to now [37]. Intracellular lipid bioavailability is a pivotal regulator of palmitoylation [24–26]. Thus, to further elucidate the precise mechanisms by which NAFLD promotes metastatic CRC stemness, we explored whether this specific protein lipidation was involved in this process. 2-bromopalmitate (2-BP) is a broad-spectrum palmitoylation inhibitor, and we found that 2-BP treatment inhibited the sphere formation capacity of primary cultured cells from metastatic CRC xenografts in a dose-dependent manner (Fig. 3E, Fig. S3A). Furthermore, 2-BP treatment decreased the expression of stemness markers in primary cultured cells from metastatic CRC xenografts (Fig. 3F). In vitro studies further confirmed that 2-BP treatment alleviated PA/OA-induced CRC cell stemness in a dose-dependent manner (Fig. 3G, H, Fig. S3B), indicating that palmitoylation might contribute to metastatic CRC cell stemness in NAFLD.

These results revealed that NAFLD might promote metastatic CRC stemness in a palmitoylation-dependent manner.

### EGFR palmitoylation critically promotes metastatic CRC cell stemness in NAFLD

**Fig. 4:**
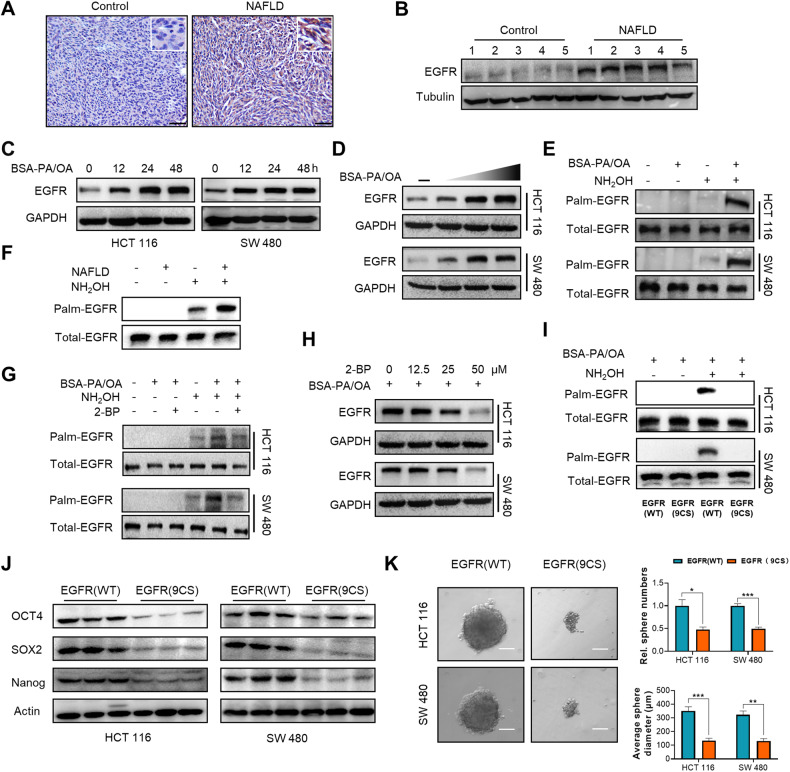
EGFR palmitoylation critically contributes to metastatic CRC cell stemness in NAFLD. **A** Representative images of IHC staining for EGFR of MC38 allografts in control and NAFLD mice. Scale bars, 50 μm. **B** The protein level of EGFR of HCT 116 xenografts in control mice and NAFLD mice by western blot assay (*n* = 5). **C** The protein level of EGFR in HCT 116 and SW 480 cells treated with or without BSA-PA/OA (PA 100 µM, OA 200 µM) at indicated time points by western blot assay. **D** The protein level of EGFR in HCT 116 and SW 480 cells treated with or without BSA-PA/OA at indicated doses for 36 h by western blot assay (-PA 0 µM, OA 0 µM; PA 50 µM, OA 100 µM; PA 100 µM, OA 200 µM; PA 200 µM, OA 400 µM, respectively). **E** The palmitoylation level of EGFR in HCT 116 and SW480 cells treated with or without BSA-PA/OA (PA 100 µM, OA 200 µM) for 24 h by acyl-biotin exchange assay. **F** The palmitoylation level of EGFR in HCT 116 xenografts in control mice and NAFLD mice by acyl-biotin exchange assay. **G** The palmitoylation level of EGFR in HCT 116 and SW480 cells treated with BSA-PA/OA (PA 100 µM, OA 200 µM) and/or 2-BP (50 µM) by acyl-biotin exchange assay. **H** The protein level of EGFR in HCT 116 and SW480 cells treated with BSA-PA/OA (PA 100 µM, OA 200 µM) and 2-BP at indicated concentrations for 24 h by western blot assay. **I** The palmitoylation level of EGFR in in control cells (EGFR-WT) and EGFR palmitoylation deficient mutant cells (EGFR-9CS) treated with BSA-PA/OA (PA 100 µM, OA 200 µM) by acyl-biotin exchange assay. **J** The protein level of CSC markers Oct4, Nanog and SOX2 in control cells (EGFR-WT) and EGFR palmitoylation deficient mutant cells (EGFR-9CS) treated with BSA-PA/OA (PA 100 µM, OA 200 µM) for 36 h by western blot assay. **K** Sphere formation assay on control cells (EGFR-WT) and EGFR palmitoylation deficient mutant cells (EGFR-9CS) treated with BSA-PA/OA (PA 100 µM, OA 200 µM). Representative photos of sphere in second passage were taken on day 10 after cells were seeded (scale bars, 100 μm), sphere numbers and diameters were determined and plotted (*n* = 3). Data are presented as mean ± SEM. Significance was determined by two-tailed unpaired Student’s *t* test, *p < 0.05, **p < 0.01, ***p < 0.001, ****p < 0.0001. BSA bovine serum albumin, PA palmitic acid, OA oleic acid, 2-BP 2-bromopalmitate.

To further explore the underlying mechanism by which NAFLD promotes metastatic CRC cell stemness via palmitoylation, we sought to preliminarily identify a potential target protein of this particular lipid modification. Palmitoylation is increasingly acknowledged as an important regulator of PM proteins [38], among which EGFR has been well recognized as a pivotal player in sustaining cancer cell stemness [39, 40] and a direct substrate protein for palmitoylation [41, 42]. Besides, EGFR was also significantly upregulated in NAFLD liver tissues and substantially contributed to disease progression [43]. Therefore, we evaluated whether palmitoylation of EGFR changed and played a critical role in NAFLD-induced liver metastatic CRC cell stemness. We firstly detected the protein expression of EGFR in metastatic CRC tissues via immunohistochemistry (IHC) and western blot assays, and the results showed that EGFR density was higher in the NAFLD group (Fig. 4A, B). In vitro studies revealed that EGFR expression was significantly upregulated in CRC cells exposed to PA/OA incubation in a time- and dose-dependent manner (Fig. 4C, D). We then conducted an acyl-biotin exchange (ABE) assay to determine whether EGFR was palmitoylated under NAFLD condition in vitro and in vivo. The ABE assay replaces palmitate on protein cysteine residues with biotin and facilitates the enrichment of palmitoylated proteins with streptavidin beads. The ABE assay results confirmed that the palmitoylation level of EGFR increased in tumor cells with PA/OA treatment in vitro and primary tumor cells isolated from NAFLD liver (Fig. 4E, F). Moreover, 2-BP treatment relieved EGFR palmitoylation and attenuated EGFR protein upregulation under NAFLD condition (Fig. 4G, H), which indicated that palmitoylation might contribute to EGFR protein upregulation in the NAFLD metabolic microenvironment. To determine whether NAFLD induced CRC cell stemness was resulted from EGFR, we knocked down EGFR in CRC cells, and the results indicated that EGFR knockdown almost completely diminished PA/OA-induced CRC cell stemness (Fig. S4A-E). Nine cysteine residues have been identified as important sites for EGFR palmitoylation, which were Cys775, Cys781, Cys797, Cys818, Cys939, Cys950, Cys1049, Cys1058, and Cys1146 [32, 42]. To further determine the contribution of EGFR palmitoylation to NAFLD microenvironment induced mCRC stemness, we constructed EGFR palmitoylation deficient mutant CRC cells (EGFR-9CS) by mutating all these nine cysteine sties to serine. ABE assays confirmed that the palmitoylation of EGFR was almost diminished in mutant cells (EGFR-9CS) (Fig. 4I). Further colony formation assay and western blot assays indicated that NAFLD induced stemness was abolished in EGFR palmitoylation deficient mutant cells (EGFR-9CS) (Fig. 4J, K, Fig. S4F, G). To strengthen our point that palmitoylated EGFR is essential to liver metastases in CRC, we further conducted liver metastasis models with EGFR palmitoylation wild-type or deficient cells in NAFLD mice. As is shown in Fig. S5, deficiency in EGFR palmitoylation greatly decreased the seeding and growth of CRC cells in the NAFLD liver.

Taken together, these results revealed that EGFR palmitoylation critically promoted metastatic CRC cell stemness and liver metastasis in NAFLD.

### Palmitoylation promotes EGFR stabilization and plasma membrane localization in metastatic CRC cells in NAFLD

**Fig. 5:**
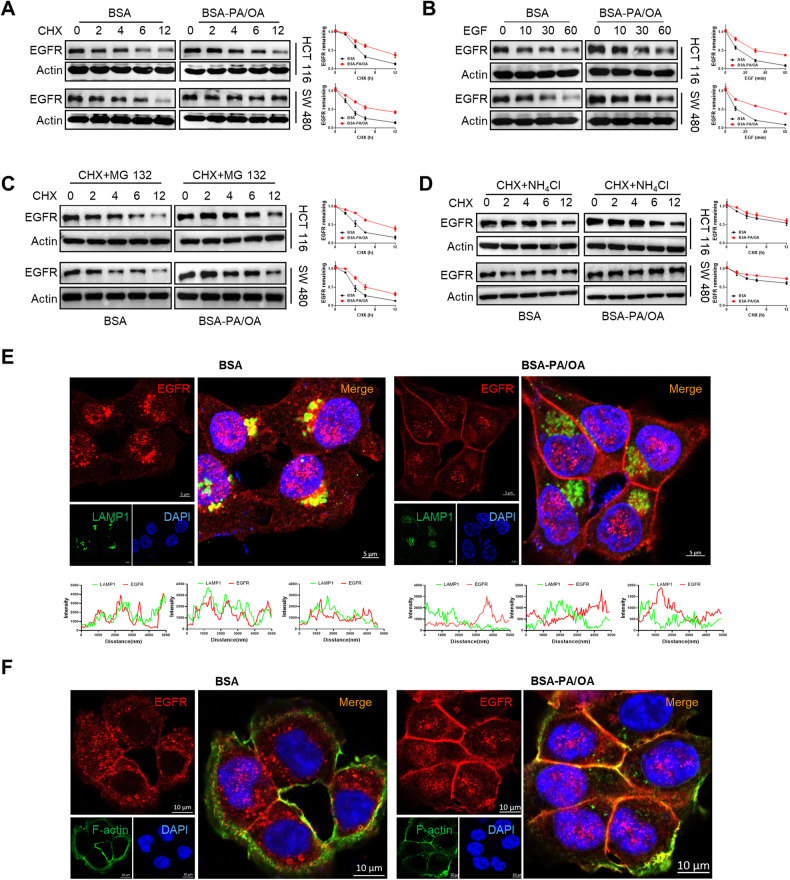
Palmitoylation promotes EGFR stabilization and plasma membrane localization in NAFLD liver metastatic CRC cells. **A** HCT 116 and SW 480 cells treated with or without BSA-PA/OA (PA 100 µM, OA 200 µM) were incubated with cycloheximide (CHX) (25 μg/ml) and analyzed by western blot at the indicated time points. Right, quantification of the results (*n* = 3). **B** HCT 116 and SW480 cells treated with or without BSA-PA/OA (PA 100 µM, OA 200 µM) were incubated with EGF (25 µg/ml) and analyzed by western blot at the indicated time points. Right, quantification of the results (*n* = 3). **C** HCT 116 and SW 480 cells treated with or without BSA-PA/OA (PA 100 µM, OA 200 µM) were incubated with 25 μg/ml CHX and 5 µM MG132 and analyzed by western blot at the indicated time points. Right, quantification of the results (*n* = 3). **D** HCT 116 and SW 480 cells treated with or without BSA-PA/OA (PA 100 µM, OA 200 µM) were incubated with 25 μg/ml CHX and 250 µM NH_4_Cl and analyzed by western blot at the indicated time points. Right, quantification of the results (*n* = 3). **E** Representative immunofluorescence images for EGFR (red), LAMP1 (green), and DAPI (blue) of HCT 116 cells treated with or without BSA-PA/OA (PA 100 µM, OA 200 µM). Scale bars, 5 μm. Intensity profiles of LAMP1 (green lines) and EGFR (red lines) co-localization signal were shown in plotted lines at three random sites. **F** Representative immunofluorescence images for EGFR (red), F-actin (green), and DAPI (blue) of HCT 116 cells treated with or without BSA-PA/OA (PA 100 µM, OA 200 µM). Scale bars, 10 μm. Data are presented as mean ± SEM. NAFLD non-alcoholic fatty liver disease, BSA bovine serum albumin, PA palmitic acid, OA oleic acid, MFI mean fluorescence intensity, 2-BP 2-bromopalmitate, FASN fatty acid synthases, CHX cycloheximide.

As palmitoylation is well acknowledged to have a profound influence on protein trafficking and stability [37], we next investigated the dynamic change of EGFR protein to gain a better understanding of the precise mechanism by which palmitoylated EGFR determined metastatic CRC cell fate in the NAFLD metabolic microenvironment. We first conducted cycloheximide (CHX) chase assays, and the results showed that after PA/OA treatment, the half-life of the EGFR protein was significantly increased (Fig. 5A). We then treated CRC cells with EGF to induce EGFR degradation, and the results revealed that EGFR expression remained barely changed after 30 min with PA/OA treatment, while EGFR expression rapidly decreased after exposure to EGF in control cells (Fig. 5B). These results indicated that PA/OA treatment delayed EGFR degradation. Moreover, the lysosome inhibitor NH_4_Cl, but not the proteasome inhibitor MG132, could greatly diminish the discrepancy in EGFR protein half-life in BSA-and BSA-PA/OA-treated CRC cells (Fig. 5C, D), suggesting that PA/OA treatment might impede the degradation of EGFR in a lysosome-dependent manner. To further validate that PA/OA decreased lysosomal degradation of EGFR, the co-localization of EGFR and the lysosome marker LAMP1 was evaluated via confocal immunofluorescence analysis. The results indicated that EGFR was less enriched in the lysosomal compartments of PA/OA-treated CRC cells (Fig. 5E). In addition, PM localization is essential for EGFR function and stability [32]. We next evaluated whether EGFR palmitoylation induced by the NAFLD metabolic microenvironment influenced its cell membrane translocation. Confocal immunofluorescence assay results showed that PM localization of EGFR was enhanced in NAFLD condition (Fig. 5F).

To further determine whether PA/OA-induced EGFR stabilization and PM localization were attributable to EGFR palmitoylation, we evaluated the effects of 2-BP and EGFR palmitoylation sites mutation (EGFR-9CS) on PA/OA-induced EGFR stabilization and PM localization. The CHX-chase assay results suggested that 2-BP and EGFR palmitoylation sites mutation (EGFR-9CS) could disrupt PA/OA-induced EGFR stabilization, and NH_4_Cl, but not MG132, rescued 2-BP induced EGFR degradation (Fig. S6A, B). In addition, confocal immunofluorescence images showed that 2-BP treatment and EGFR palmitoylation sites mutation (EGFR-9CS) increased EGFR localization in lysosomes and reduced EGFR PM localization (Fig. S6C-F). These results indicated that inhibiting palmitoylation promoted EGFR lysosomal degradation and suppressed its PM localization in NAFLD.

Based on these evidences, we demonstrated that the NAFLD metabolic microenvironment promoted EGFR palmitoylation and that this protein lipid modification might promote its localization in PM and evasion from lysosomal degradation.

### NAFLD activates endogenous palmitate biosynthesis in metastatic CRC cells to facilitate EGFR palmitoylation

**Fig. 6:**
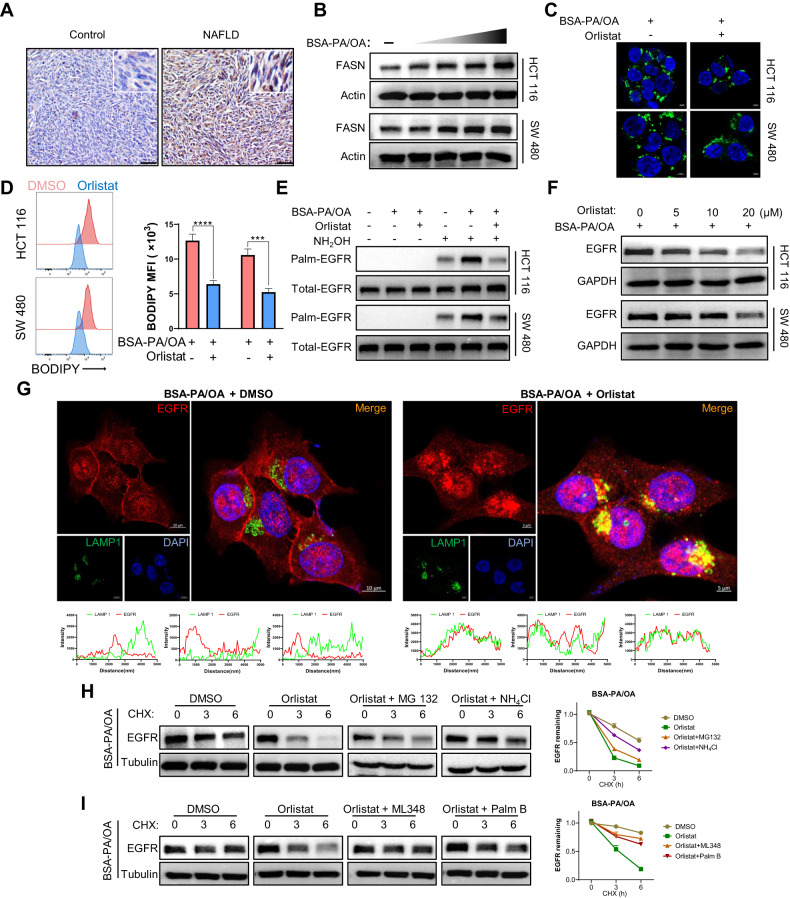
NAFLD promotes EGFR palmitoylation via activating de novo palmitate biosynthesis in metastatic CRC cells. **A** Representative images of IHC staining for FASN of MC38 allografts in control and NAFLD mice. Scale bars, 50 μm. **B** The protein level of FASN in HCT 116 and SW 480 cells treated with or without BSA-PA/OA at indicated doses (PA 0 µM, OA 0 µM; PA 50 µM, OA 100 µM; PA 100 µM, OA 200 µM; PA 200 µM, OA 400 µM, respectively) by western blot assay. **C** Representative fluorescence imaging of BODIPY 493/503 staining (green) in HCT 116 and SW 480 cells treated with BSA-PA/OA (PA 100 µM, OA 200 µM) and/or 10 µM orlistat. Nuclei were stained with DAPI (blue). **D** Representative quantitative plots of the histogram (left) and MFI (right) of BODIPY 493/503 staining in BSA-PA/OA (PA 100 µM, OA 200 µM) incubated HCT 116 and SW 480 cells treated with or without 10 µM orlistat. **E** The palmitoylation level of EGFR in HCT 116 and SW480 cells treated with or without BSA-PA/OA (PA 100 µM, OA 200 µM) and 10 µM orlistat by acyl-biotin exchange assay. **F** The protein level of EGFR in HCT 116 and SW480 cells treated with BSA-PA/OA (PA 100 µM, OA 200 µM) and indicated concentrations of orlistat by western blot assay. **G** Representative immunofluorescence images for EGFR (red), LAMP1 (green), and DAPI (blue) of BSA-PA/OA (PA 100 µM, OA 200 µM) treated HCT116 cells incubated with or without 10 µM orlistat. Scale bars, 10 μm. Intensity profiles of LAMP1 (green lines) and EGFR (red lines) co-localization signal were shown in plotted lines at three random sites. **H** Left, the degradation of EGFR treated with BSA-PA/OA (PA 100 µM, OA 200 µM) in HCT 116 cells was evaluated by CHX-chase assay in the presence of orlistat and/or inhibitors for proteasome MG132 and lysosome NH_4_Cl. Right, quantification of the intensity determined by the relative level of EGFR remaining (*n* = 3). **I** Left, the degradation of EGFR treated with BSA-PA/OA (PA 100 µM, OA 200 µM) in HCT 116 cells was evaluated by CHX-chase assay in the presence of orlistat and/or inhibitors for depalmitoylation ML348, Palm B. Right, quantification of the intensity determined by the relative level of EGFR remaining (*n* = 3). Data are presented as mean ± SEM. Significance was determined by two-tailed unpaired Student’s *t* test, *p < 0.05, **p < 0.01, ***p < 0.001, ****p < 0.0001. NAFLD non-alcoholic fatty liver disease, BSA bovine serum albumin, PA palmitic acid, OA oleic acid, MFI mean fluorescence intensity, 2-BP 2-bromopalmitate, FASN fatty acid synthases, CHX cycloheximide.

As is revealed that NAFLD metabolic microenvironment enriched lipid bioavailability in metastatic CRC cells and promoted metastatic CRC cell stemness via EGFR palmitoylation, we then sought to identify a clinically available therapeutic approach to disturb this process and suppress liver metastasis of CRC in NAFLD. The sources of intracellular lipid content are generally determined by both exogenous lipids uptake and endogenous de novo lipogenesis (DNL) [18]. Aberrant hepatic lipid accumulation is a canonical characteristic of the NAFLD metabolic microenvironment [44], thus, it is reasonable to assume that inhibiting exogenous lipid uptake could be a promising way to disrupt the metabolic crosstalk between the NAFLD microenvironment and liver metastatic CRC cells. Thus far, the membrane protein CD36 has been best justified as a transporter of fatty acids [45], and a western blot assay also suggested that CD36 expression was upregulated in metastatic CRC liver tissues from NAFLD mice, making CD36 a rational target (Fig. S7A). Unfortunately, there are currently no approaches to inhibit CD36 mediated lipid uptake specifically and efficiently for clinical use. Therefore, we further determined whether and how DNL in metastatic CRC cells was remodeled in the NAFLD metabolic microenvironment. We then detected the expression of de novo lipogenic enzymes in metastatic CRC tissues, and found that DNL was enhanced (Fig. S7B-C). FASN is the key enzyme that directly mediates the endogenous biosynthesis of palmitate, and further IHC assay of metastatic CRC allograft tissues showed that FASN expression was significantly upregulated in NAFLD mice (Fig. 6A). As shown in Fig. 6B, the protein level of FASN in CRC cells was significantly upregulated in a concentration-dependent manner following PA/OA treatment in vitro. These results indicate that endogenous de novo palmitate synthesis is upregulated and potentially contributes to lipid bioavailability enrichment in metastatic CRC cells in NAFLD. Orlistat is an FDA-approved anti-obesity drug and its target was identified as FASN. We aimed to determine whether orlistat could decrease intracellular lipid content and induce EGFR lysosomal degradation and PM delocalization in NAFLD. The BODIPY staining results indicated that orlistat decreased the intracellular lipid bioavailability of CRC cells under NAFLD condition (Fig. 6C, D). The ABE assay results indicated that orlistat could inhibit EGFR palmitoylation under NAFLD condition (Fig. 6E). Western blotting results showed that orlistat decreased EGFR expression in a dose-dependent manner following PA/OA treatment (Fig. 6F). Further confocal immunofluorescence results confirmed that orlistat promoted EGFR and lysosome co-localization (Fig. 6G) and decreased EGFR PM localization under PA/OA treatment (Fig. S7D). The lysosome inhibitor NH_4_Cl also rescued orlistat-induced EGFR degradation (Fig. 6H). To further elucidate whether orlistat could decrease EGFR expression by inhibiting its palmitoylation, we treated CRC cells with depalmitoylation inhibitors ML348 and Palm B to enhance its palmitoylation. CHX-chase assay results indicated that Palm B and ML348 delayed EGFR degradation induced by orlistat in NAFLD conditions (Fig. 6I).

Altogether, these results revealed that the FDA-approved drug orlistat could decrease EGFR palmitoylation and suppress its PM localization and evasion from lysosomal degradation in NAFLD.

### Orlistat suppresses NAFLD induced CRC cell stemness and restricts liver metastasis in synergy with chemotherapy

**Fig. 7:**
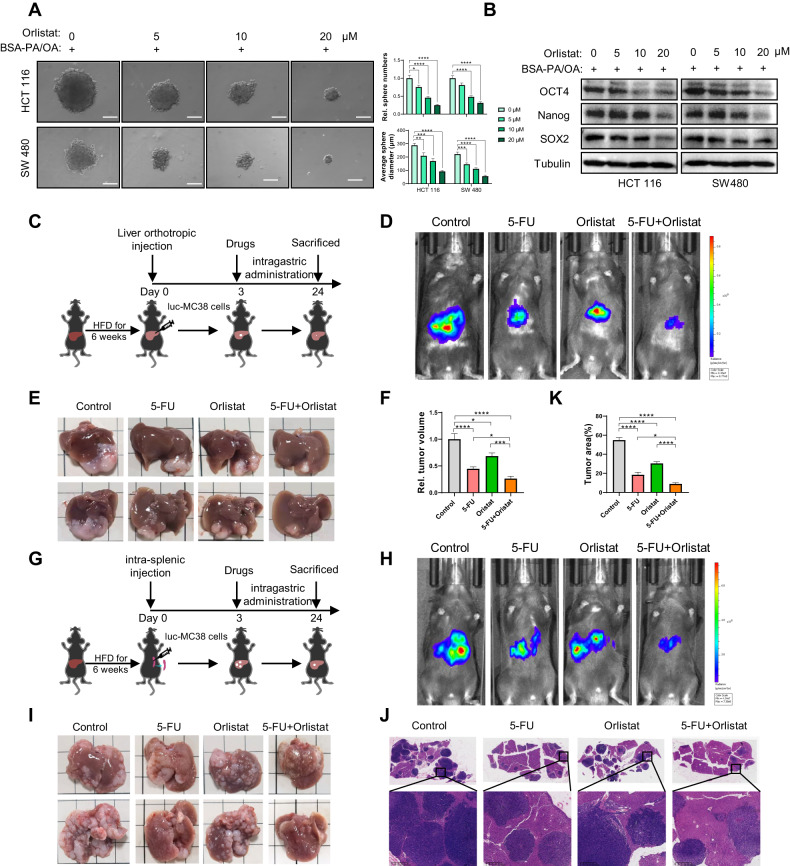
Orlistat suppresses CRC cell stemness and restricts liver metastasis synergized with chemotherapy in NAFLD. **A** Sphere formation assay in BSA-PA/OA (PA 100 µM, OA 200 µM) treated HCT 116 and SW 480 cells incubated with or without orlistat at indicated doses. Representative photos were taken on Day 10 after seeding in second passage, scale bar represents 100 µm. Sphere numbers and diameters were determined and plotted (*n* = 3). **B** The protein level of CSC markers Oct4, Nanog and SOX2 in BSA-PA/OA (PA 100 µM, OA 200 µM) treated HCT 116 and SW 480 cells incubated with or without orlistat at indicated concentrations by western blot assay. **C**–**F** Schematics illustrating the experimental liver metastasis model established via liver orthotopic implantation in C57BL/6 mice (**C**). In vivo bioluminescent images of luc-MC38 in the livers of mice treated with orlistat and/or 5-FU or the vehicle control in intrahepatic injection liver metastasis models (**D**). At the end of the experiment, liver metastases were taken photos and assess metastatic lesion volume, and plotted (**E**, **F**). **G**–**K** Schematics illustrating the experimental liver metastasis model established via splenic implantation in C57BL/6 mice (**G**). In vivo bioluminescent images of luc-MC38 in the livers of mice treated with orlistat and/or 5-FU or the vehicle control in splenic injection liver metastasis models (**H**). At the end of the experiment, liver metastases were harvested for taking photos, H&E staining and assessing tumor area (**I**–**K**). Data are presented as mean ± SEM. Significance was determined by one-way ANOVA, *p < 0.05, **p < 0.01, ***p < 0.001, ****p < 0.0001. BSA bovine serum albumin, PA palmitic acid. OA oleic acid, 5-FU fluorouracil, HFD high-fat diet, luc-MC38 cells luciferase-labeled MC38 cells.

We then evaluated the effects of orlistat on the suppression of CRC cell stemness in NAFLD. The results indicated that orlistat reduced sphere formation capacity and decreased PA/OA-induced stemness marker upregulation in a dose-dependent manner (Fig. 7A, B, Fig. S8A, B). To further evaluate the effects of orlistat in suppressing CRLM enhanced by NAFLD in vivo, we established NAFLD mice models with HFD for 6 weeks and then implanted murine CRC cell MC38 via the liver and spleen to mimic CRLM (Fig. 7C, G). The standard first-line chemotherapy regimen for liver metastatic CRC comprises 5-fluorouracil (5-FU), and the effects of orlistat in combination with 5-FU were also evaluated. Three days after implantation, the mice were divided into four groups and treated with PBS, 5-FU, orlistat, and 5-FU combined with orlistat respectively. After 3 weeks, in vivo bioluminescent imaging was conducted, and the results indicated that both hepatic CRC colonization and growth were remarkably reduced in 5-FU- and orlistat-treated mice compared to those in control mice, and orlistat had a synergistic effect with 5-FU (Fig. 7D, H). The liver was harvested to evaluate CRC metastasis, and the results showed that there was a significant decrease in both the number and size of metastatic tumor nodules in the livers of 5-FU- and orlistat-treated mice, and orlistat greatly enhanced the effects of 5-FU (Fig. 7E, F, I-K). These results indicated that orlistat could inhibit metastatic CRC stemness and suppress liver metastasis in synergy with conventional chemotherapy in NAFLD.

## Discussion

Liver metastasis of CRC remains a critical clinical challenge. About 10–15% of CRC patients have synchronous liver metastases at initial diagnosis, and almost half of patients with CRC would eventually develop liver metastasis during disease progression, whereas only 10–20% of patients with liver metastatic CRC can undergo surgical resection [46]. Liver metastases of CRC are mainly attributed to the portal venous system, which connects the colorectum and liver with abundant bloodstream. It has been recently discovered that the addiction of CRC to various liver metabolites, such as fructose [47], also contributed to its metastatic tropism to the liver. The liver is the principal organ for systemic lipid metabolism. Lipid metabolites are especially copious in the hepatic microenvironment, and lipid abundance is especially amplified when NAFLD occurs. Recent reports have revealed that lipids could promote CRC progression by upregulating β2-adrenergic receptor and toll-like receptor 4 (TLR4) expression [48, 49] and could activate the STAT3 signaling pathway in prostate cancer as a signal transduction mediator [50]. A previous study indicated that the stimulated liver metastasis of melanoma and breast cancer in NAFLD was mediated by enhanced lipid transfer from hepatocytes to liver metastatic melanoma and breast cancer cells, where lipids were metabolized as energy sources by mitochondrial oxidation to fuel metastatic tumor growth [9]. In this study, we highlight lipids as pivotal post-translational modification substrates that promote EGFR protein stability and PM localization in a palmitoylation-dependent manner and enhance metastatic cancer cell stemness beyond their original functions as energy sources and signaling mediators. Besides CRC, liver is the most prevalent distant metastatic niche for other cancers as well, such as pancreatic ductal adenocarcinoma, gastric cancer and breast cancer. It is also noteworthy to investigate whether the NAFLD metabolic microenvironment-induced palmitoylation-dependent alterations also occur and drive liver metastasis of these cancers.

The liver metastatic tumor microenvironment (TME) consists of multiple immune cells, including T cells, B cells, macrophages, and NK cells, along with fibroblasts and the extracellular matrix, which coordinate to form a particular niche for the seeding and expansion of metastatic tumor cells in the liver. Recently, excess lipid deposits in NAFLD were reported to upregulate NLRC4 expression, a crucial component of the inflammasome, via activating TLR4 in tumor-associated macrophages in the liver, promoting their polarization towards M2 and enhancing IL-1β and VEGF production to favor the growth of metastatic CRC cells [10]. However, the effects of the NAFLD metabolic microenvironment on other TME components are still elusive, and it will be of particular interest to explore the roles of these TME components in NAFLD-promoted CRLM and illustrate their respective mechanisms. Palmitoylation, as the most well-established lipid modification, also has a profound influence on immune cells. There are multiple pivotal targets in immune cells that are known to be substrates of palmitoylation. cGAS was found to be palmitoylated, which could negatively regulate innate immunity [51], and the palmitoylation status of STAT3 could determine Th17 cell differentiation [52]. Palmitoylation of NOD1/2 is required for membrane recruitment and immune signaling in macrophages [53]. Thus, it could be of significance to determine whether palmitoylation-dependent alterations also exist in these immune cells and influence liver metastasis of CRC and their response to immune therapy in NAFLD in future research.

Recent reports have revealed that systemic metabolism significantly influences cancer cell metabolism [13, 54]. As for primary tumors, the tumor metabolic microenvironment is determined by the cellular metabolism of tumor cells and other TME components, metabolic interactions between tumor and non-tumor cells, and their communication with the systemic metabolism of the whole body [55]. As revealed by Ringel et al. that obesity as a systemic metabolic stress converts the tumor metabolic microenvironment by inducing competition between cancer cells and T cells for lipids, and blocking this obesity-induced metabolic adaptation could evoke intense antitumor immune responses in obese cancer patients [56]. In terms of metastasis, metastatic cancer cells intended to switch their intrinsic metabolic patterns towards that of their colonized organs under the influence of the metastatic metabolic microenvironment [57]. The liver is a major organ for systemic cholesterol and fructose metabolism, and recent studies have indicated that liver metastatic CRC cells have increased fructose and cholesterol metabolism, and genetically or pharmacologically inhibiting these metabolic switches could suppress liver metastasis of CRC [47, 58]. NAFLD as a systemic metabolic stress enhanced DNL in hepatocytes via multiple mechanisms, such as upregulating of USP14 to deubiquitinate FASN and promote its stability, and depleting SH3RF2 to stabilize ATP citrate lyase (ACLY) [59, 60]. In this study, we found that the NAFLD hepatic metabolic microenvironment could also enhance de novo palmitate biosynthesis in liver metastatic CRC cells and that enriched intracellular palmitate bioavailability could promote cancer cell stemness by facilitating palmitoylation of EGFR to promote its stability and PM localization. EGFR has previously been reported to be a substrate protein for palmitoylation by several studies [32, 41]. In accordance with our findings, previous studies have suggested that inhibiting EGFR palmitoylation with 2-BP could decrease EGFR PM localization, reduce EGFR protein expression, and sensitize lung and breast cancer cells to EGFR-targeted tyrosine kinase inhibitors (TKIs) treatments [41, 61]. Taken together, these findings demonstrate that targeting the palmitoylation of EGFR represents a promising strategy for tumors harboring wild-type EGFR. Nevertheless, 2-BP is a general palmitoylation inhibitor with severe off-target toxicity; therefore, a safe and efficient therapeutic approach to inhibit EGFR palmitoylation for clinical use is still not available. In this study, we found that using an FDA-approved de novo palmitate synthesis inhibitor, orlistat, to block NAFLD induced metabolism adaptation could inhibit EGFR palmitoylation and suppress CRLM in NAFLD, and orlistat treatment has a synergistic effect with conventional chemotherapy. Currently, there are no FDA-approved pharmacological interventions for clinical NAFLD treatment, and our study provided proof-of-concept evidences for the potential use of orlistat in suppressing liver metastasis and sheds light on the improvement of prognosis of CRC patients with NAFLD.

**Fig. 8:**
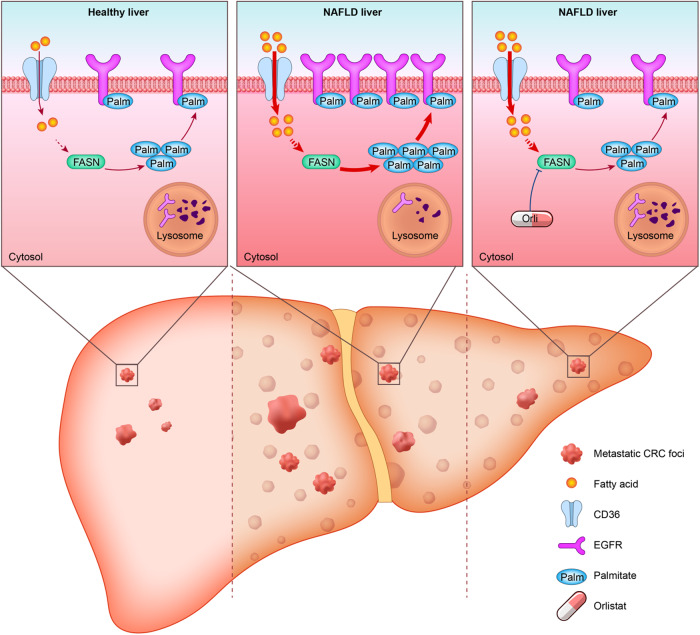
Schematic of the proposed working model. The NAFLD metabolic microenvironment activates endogenous palmitate biosynthesis in liver metastatic CRC cells, leading to palmitoylation-dependent EGFR stabilization and plasma membrane localization. Inhibition of FASN with orlistat suppresses NAFLD-driven CRC liver metastasis.

In conclusion, we demonstrate that NAFLD enhances de novo palmitate synthesis in metastatic CRC cells and promotes CRLM via palmitoylation-dependent EGFR stabilization and PM localization, and that orlistat could be a promising adjuvant drug for suppressing NAFLD-induced CRLM and has synergistic effects with chemotherapy (Fig. 8).

## Materials and methods

### Cell culture and reagents

Human CRC cell lines HCT 116 (RRID: CVCL_0291) and SW480 (RRID: CVCL_0546), and the murine CRC cell line MC38 (RRID: CVCL_B288) were cultured in DMEM supplemented with 10% FBS. All cell lines were obtained from the American Type Culture Collection (ATCC; Manassas, Virginia, USA) and incubated with 5% CO_2_ at 37 °C. All cell lines were routinely tested for mycoplasma contamination and authenticated using short tandem repeat. To stably label HCT 116 and MC38 cells, a lentiviral vector containing luciferase reporter genes was used. 2-bromopalmitate, ML348, Palm B, orlistat, NH_4_Cl, MG132, and cycloheximide were administered at the indicated concentrations.

### Animal studies

Male mice aged 8–12 weeks were fed either a low-fat diet (LFD; 12% calories from fat) or a high-fat diet (HFD; 60% calories from fat) for 6 weeks. All mice were divided into five mice per cage and reared in independently vented cages at the animal facility of the Laboratory Animal Center, Southwest Hospital. We determined sample size with an animal sample size calculator downloaded from https://intraweb.hku.hk/local/launit/content/forms/Animal_Sample_Size_Calculations_for_Anticipated_Means_or_from_Pilot_Study_or_Publication_Data.xlsx. To establish an intrasplenic injection model, HCT 116 cells (2 × 10^6^ in 100 μL PBS) or MC38 cells (5 × 10^5^ in 100 μL PBS) were inoculated into the spleen. A liver orthotropic injection model was created by injecting HCT 116 cells (2 × 10^6^ in 50 μL PBS) or MC38 cells (5 × 10^5^ in 50 μL PBS) into the mouse liver capsule. Two weeks post-injection (for a total of 8 weeks of HFD feeding), tumor growth was monitored by a bioluminescence assay (IVIS, Caliper Sciences), and the livers were harvested and analyzed. For the drug treatment experiments, 3 days after injection, mice were randomly divided into four groups (five mice in each group) and administered vehicle control, orlistat (10 mg/kg/day) intragastrical daily, or 5-Fluorouracil (5-FU; 15 mg/kg/day) intraperitoneal every three days. At the end of each session (~3 weeks), the mice were sacrificed and their livers were dissected and photographed. All experiments were performed with 8–12 weeks-old male C57BL/6 mice (RRID: MGI:2159769) and nude mice (BALB/c nu/nu, RRID: MGI:5652590), obtained from Hunan SJT Laboratory Animal Co. Ltd. (Hunan, China) and maintained at 22 °C with a 12-h light-dark cycle. All experiments involving animals were performed in accordance with ethical policies and procedures approved by the Experimental Animal Welfare and Ethics Committee of the Third Military Medical University.

### Acyl-biotin exchange (ABE) assay

Cell samples were lysed with lysis buffer, added with 1X protease inhibitor cocktail (Roche) and 5 mM PMSF (Beyotime). Cell lysates were then determined for protein concentration with the BCA kit (Beyotime) and prepared to 2 mg/mL with lysis buffer. Then, 200 μg of total protein in 92.5 μL of lysis buffer was added with 5 μL of 200 mM neutralizing TCEP (Beyotime) to a final concentration of 10 mM for half an hour with nutation. Next, 2.5 μL of 1000 mM NEM (Sigma) was incorporated to a final concentration of 25 mM and incubated for 2 h at room temperature with nutation. Methanol/chloroform protein precipitation was used to extract protein pellets. The protein pellet was afterwards resuspended in 30 μL of PBS buffer containing 4% SDS, 4 mM EDTA and added with 90 μL of 1 M neutralizing NH_2_OH, dissolved in pH 7.3 PBS buffer containing 0.2% Triton X-100, to gain a final concentration of 0.75 M NH_2_OH. Control samples were diluted in 90 µL PBS buffer containing 0.2% Triton X-100. Incubate the samples at room temperature for 1 h while nutating. The samples were then subjected to methanol/chloroform precipitation. Prepare 0.5 ml of Biotin-BMCC buffer per sample at a working concentration between 0.5 μM and 5 μM. Add 0.5 ml of Biotin-BMCC buffer to each sample and nutate at 4° for 1 h. The samples were then subjected to methanol/chloroform precipitation. Incubate the protein with streptavidin for 2 h at 4 °C. After washed with PBST, the immunoprecipitants were eluted, neutralized, and denatured by SDS for further immunoblotting [62].

### Extreme limiting dilution assay (ELDA)

Extreme limiting dilution assay was performed as described [63, 64]. Different dilutions (1, 10, 25, 50 cells/well) for HCT 116 cells were seeded in 96-well plates and treated with BSA or BSA-PA/OA (PA 100 µM, OA 200 µM) respectively. After 2 weeks, the number of spheres formed was counted and analyzed using an online software application (http://bioinf.wehi.edu.au/software/elda/).

### Materials

Detailed information on the antibodies and other reagents used is provided in Supplementary Table 1. The shRNA sequences are listed in Supplementary Table 2. Additional methods have been described in the Supplementary Methods section.

### Statistical analysis

Data are presented as the mean ± standard deviation or mean ± standard error of the mean of at least three independent experiments. All statistical analyses were performed using IBM SPSS Statistics for Windows (RRID: SCR_002865) version 13.0. Student’s *t* test was used to test the significance of the differences between the two groups. One-way analysis of variance (ANOVA) was used for multigroup comparisons. Data were analyzed using the GraphPad Prism Software (RRID: SCR_002798). A value of *p* < 0.05 was considered statistically significant. In all experiments, animals were randomly assigned to different treatment groups without blinding, and sample sizes are specified in each figure legend. No samples or animals were excluded from our analyses. For every figure, appropriate statistical tests were applied, and all data met the assumptions of these tests. Although we did not use statistical methods to predetermine sample sizes, our sample sizes are consistent with those commonly used in the field.

### Supplementary information

Supplemental material

Original Data File

## Data Availability

All data generated or analyzed during this study are included in this published article and its supplementary information files.
