# Supplementary material for: Non-alcoholic fatty liver disease promotes liver metastasis of colorectal cancer via fatty acid synthase dependent EGFR palmitoylation
Source: Cell Death Discov. 2024 Jan 23;10:41. doi: 10.1038/s41420-023-01770-x (PMC10805926; doi:10.1038/s41420-023-01770-x)
Supplement: Supplementary file 1 — Supplemental material [file 41420_2023_1770_MOESM1_ESM.pdf]

Supplementary Materials

Supplementary figures

Figure S1

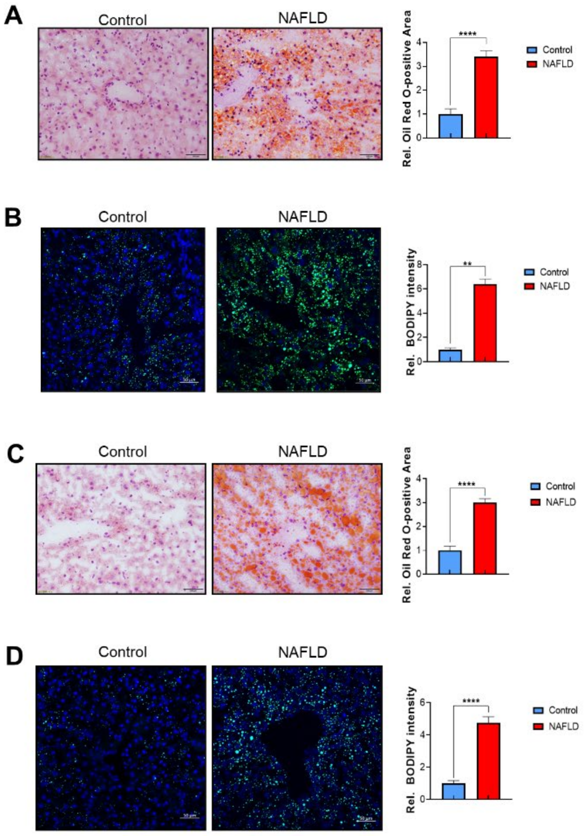

Figure S1 NAFLD is successfully induced in C57BL/6 and BALB/c nude mice models. (A) Representative imaging of lipid droplets stained with Oil Red O, and corresponding quantification data in livers in control and NAFLD C57BL/6 mice (n=5). scale bars, 100 μm. (B) Representative fluorescence imaging of lipid droplets stained with BODIPY 493/503 (green), and corresponding quantification data in livers in control and NAFLD C57BL/6 mice (n=5). Nuclei were stained with DAPI (blue). scale

bars, 50 μm. (C) Representative imaging of lipid droplets stained with Oil Red, and corresponding quantification data in in livers in control and NAFLD BALB/c nude mice (n=5). scale bars, 100 μm. (D) Representative fluorescence imaging of lipid droplets stained with BODIPY 493/503 (green), and corresponding quantification data in livers in control and NAFLD BALB/c nude mice (n=5). Nuclei were stained with DAPI (blue). scale bars, 50 μm. Data are presented as mean ± SE mean. Significance was determined by two-tailed unpaired Student's *t* test, *p < 0.05, **p < 0.01, ***p < 0.001. NAFLD, non-alcoholic fatty liver disease; No., number.

Figure S2

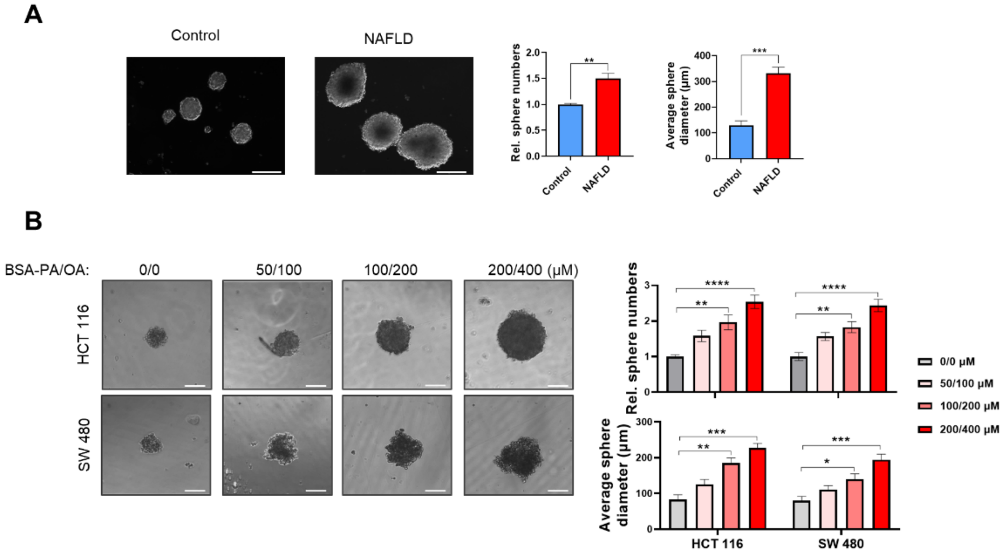

Figure S2 NAFLD metabolic niche promotes CRC cell stemness *in vitro*. (A) Sphere formation assay on tumour cells isolated from HCT 116 xenografts in control and NAFLD mice. Representative photos of HCT 116 sphere in the first passage were taken on day 7 after cells were seeded (scale bars, 200 μm), and sphere numbers and diameters

were determined and plotted (n=3). (B) Sphere formation assay on HCT 116 and SW 480 cells treated with BSA-PA/OA at indicated concentrations (PA 0 μM, OA 0 μM; PA 50μM, OA 100μM; PA 100μM, OA 200μM; PA 200μM, OA 400μM, respectively). Representative photos of sphere in the first passage were taken on day 7 after cells were seeded (scale bars, 100 μm), and sphere numbers and diameters were determined and plotted (n=3). Data are presented as mean ± SE mean. Significance was determined by two-tailed unpaired Student's *t* test (A) and one-way ANOVA (B), $*p < 0.05$, $**p < 0.01$, $***p < 0.001$. NAFLD, non-alcoholic fatty liver disease; BSA, bovine serum albumin; PA, palmitic acid; OA, oleic acid.

Figure S3

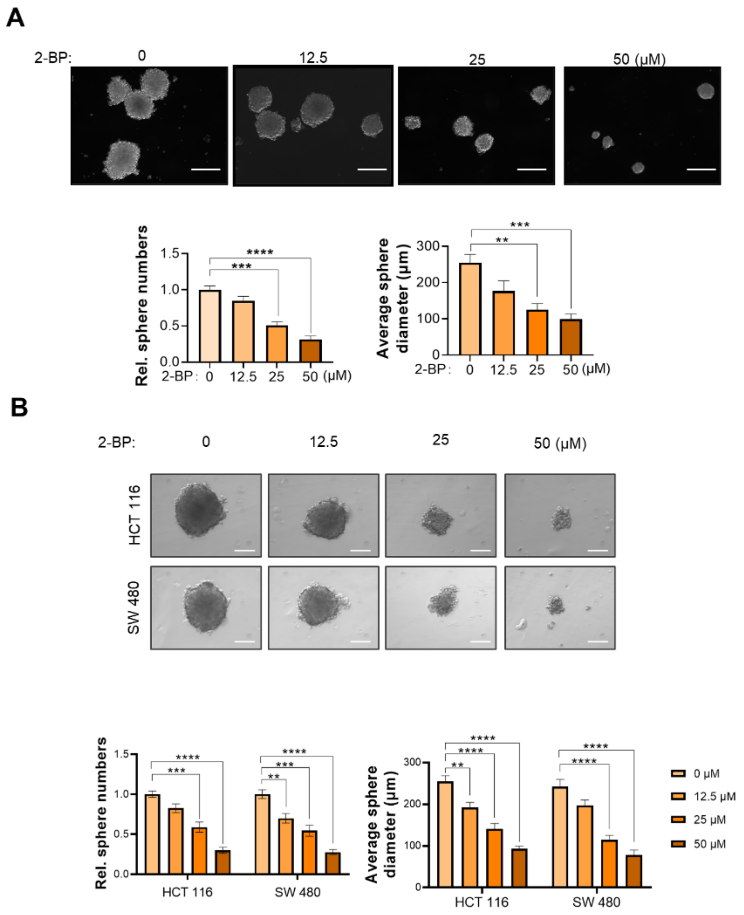

Figure S3 Inhibiting palmitoylation suppresses CRC cell stemness in NAFLD metabolic microenvironment. (A) Sphere formation assay on tumour cells isolated from HCT116 allografts in control and NAFLD mice and exposed to indicated concentrations of 2-BP. Representative photos of sphere in first passage were taken on day 7 after cells were seeded (scale bars, 200 μm), and sphere numbers and diameters were determined and plotted (n=3). (B) Sphere formation assay in HCT 116 and SW480 cells treated with or without 2-BP at indicated doses. Representative photos of sphere in first passage were taken on day 7 after cells were seeded (scale bars, 100 μm), and sphere numbers and diameters were determined and plotted (n=3). Significance was

determined by one-way ANOVA, *p < 0.05, **p < 0.01, ***p < 0.001. 2-BP, 2-bromopalmitate.

Figure S4

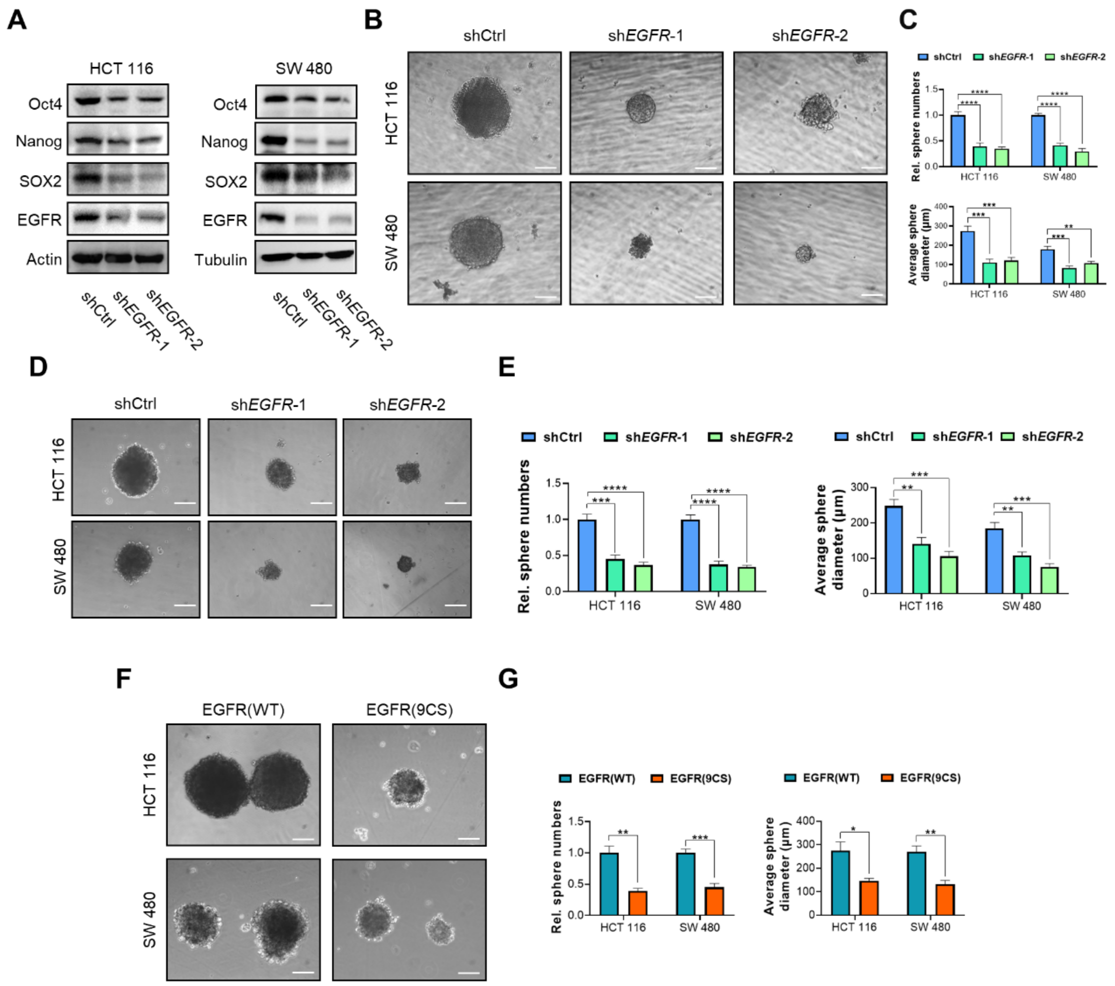

Figure S4 EGFR is essential for CRC cell stemness in NAFLD. (A) The protein level of EGFR and CSC markers Oct4, Nanog and SOX2 in control cells and EGFR KD cells treated with BSA-PA/OA (PA 100 μM, OA 200 μM) for 36h by western blot assay. (B-C) Sphere formation assay on control cells and EGFR KD cells treated with BSA-

PA/OA (PA 100 μM, OA 200 μM). Representative photos of sphere in second passage were taken on day 10 after cells were seeded (scale bars, 100 μm), sphere numbers and diameters were determined and plotted (n = 3). (D-E) Sphere formation assay on control cells and EGFR KD cells treated with BSA-PA/OA (PA 100μM, OA 200μM). Representative photos of sphere in first passage were taken on day 7 after cells were seeded (scale bars, 100 μm). Sphere numbers and diameters were determined and plotted (n=3). (F-G) Sphere formation assay on control cells (EGFR-WT) and EGFR-palmitoylation deficient mutant cells (EGFR-9CS) treated with BSA-PA/OA (PA 100 μM, OA 200 μM). Representative photos of sphere in first passage were taken on day 7 after cells were seeded (scale bars, 100 μm), sphere numbers and diameters were determined and plotted (n = 3). Data are presented as mean ± SEM. Significance was determined by one-way ANOVA (C, E) and two-tailed unpaired Student's *t* test (G), *$p < 0.05$, **$p < 0.01$, ***$p < 0.001$, ****$p < 0.001$.

Figure S5

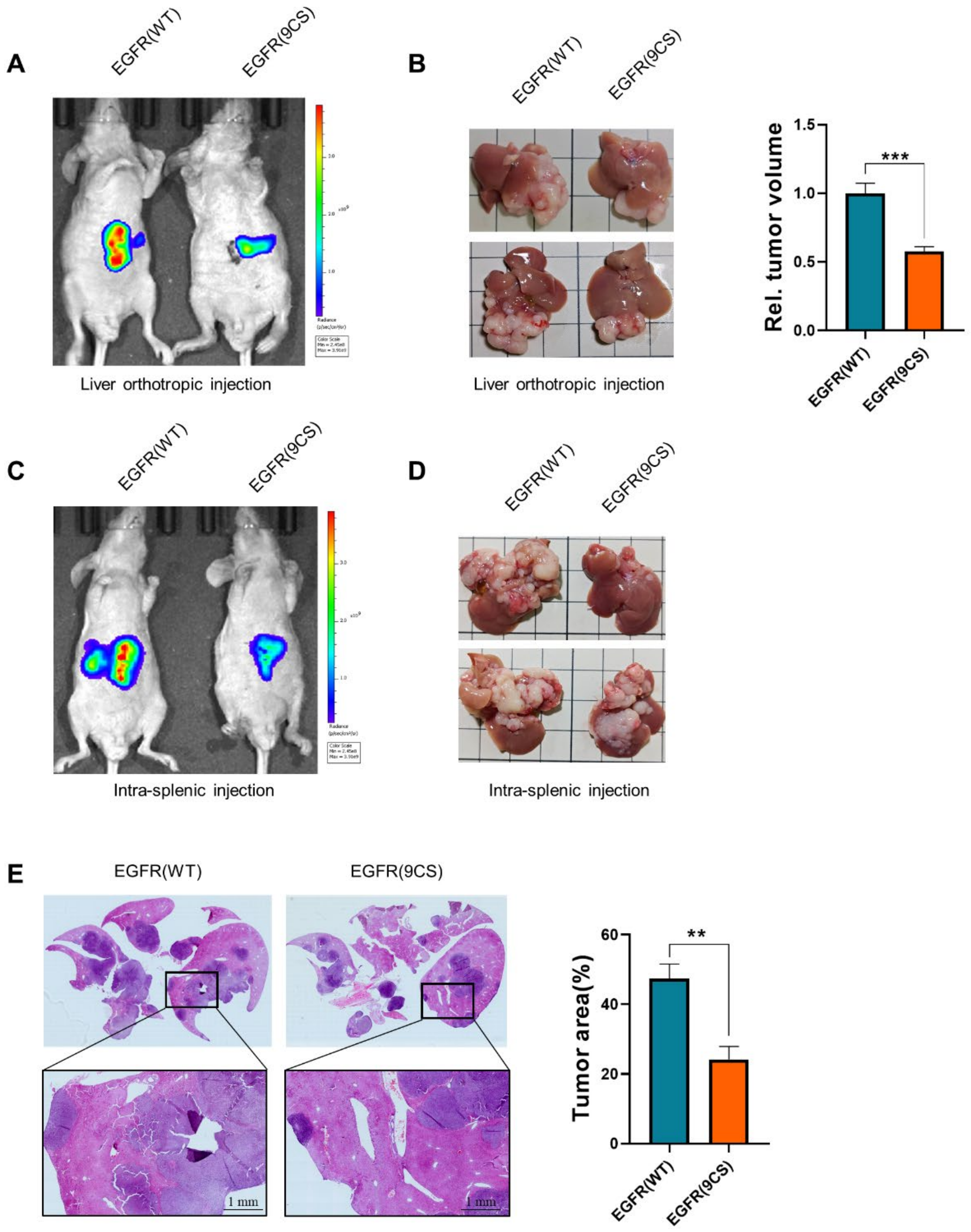

Figure S5 Palmitoylated EGFR is essential to CRC liver metastases in NAFLD liver. (A) *In vivo* bioluminescent images of EGFR(WT) or EGFR(9CS) luc-HCT116 in the livers of NAFLD in intrahepatic injection liver metastasis models. (B) At the end of the experiment, liver metastases were taken photos, assessed metastatic lesion volume, and plotted. (C) *In vivo* bioluminescent images of EGFR(WT) or EGFR(9CS) luc-HCT116 in the livers of NAFLD in splenic injection liver metastasis models. (D) At the

end of the experiment, liver metastases were harvested for taking photos. (E) H&E staining and assessing tumor area. Data are presented as mean ± SEM. Significance was determined by two-tailed unpaired Student's *t* test, *$p < 0.05$, **$p < 0.01$, ***$p < 0.001$, ****$p < 0.0001$.

Figure S6

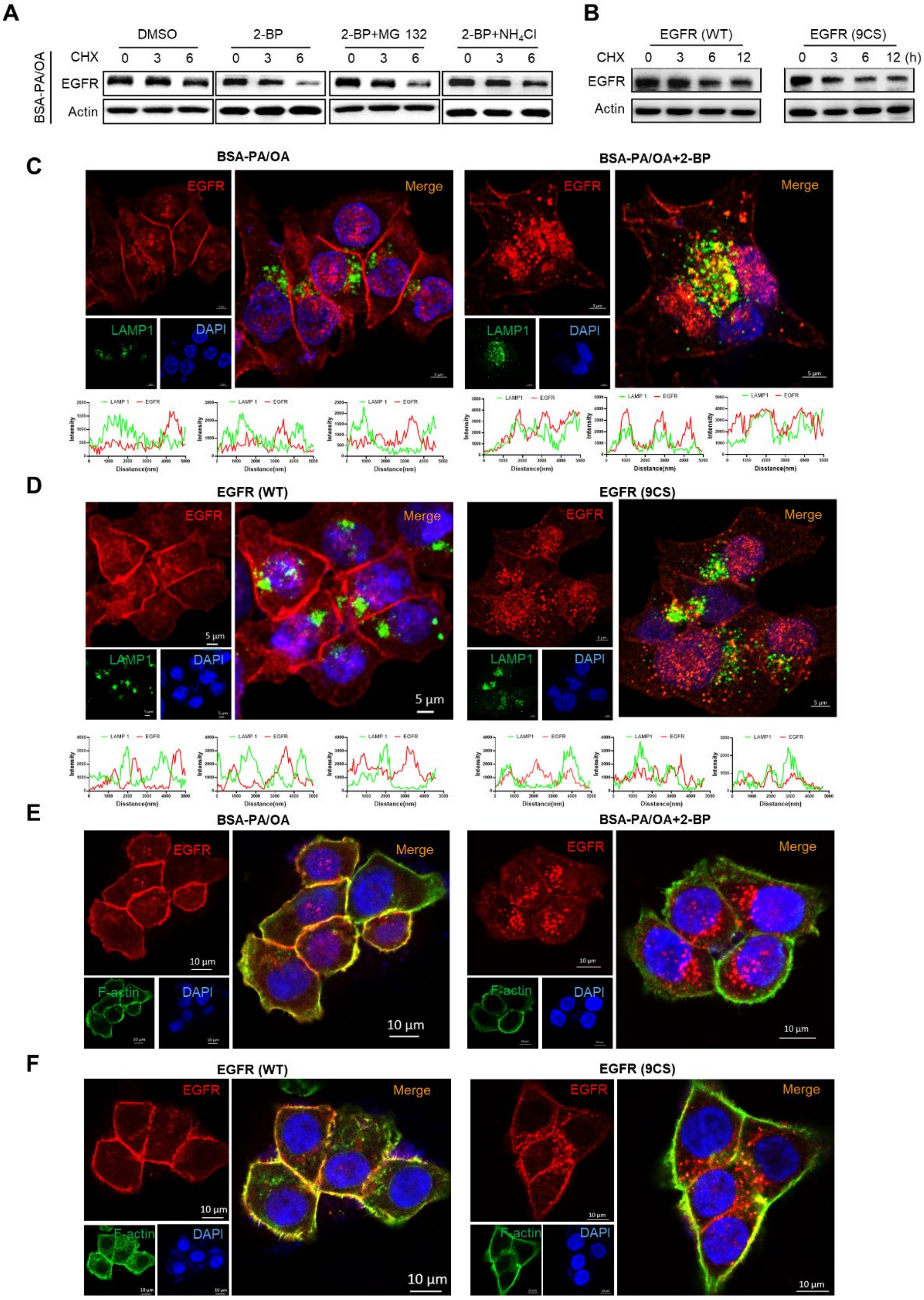

Figure S6 Inhibiting palmitoylation promotes EGFR destabilization and plasma membrane delocalization. (A) The degradation of EGFR treated with BSA-PA/OA

(PA 100 μM, OA 200 μM) in HCT 116 cells was evaluated by CHX-chase assay in the presence of inhibitors for palmitoylation (2-BP), proteasome (MG132), and lysosome (NH_4Cl). (B) The degradation of EGFR in control (EGFR-WT) and EGFR palmitoylation-deficient HCT 116 cells (EGFR-9CS) was evaluated by CHX chase assay. (C) Representative immunofluorescence images for EGFR (red), LAMP1 (green), and DAPI (blue) of BSA-PA/OA (PA 100 μM, OA 200 μM) treated HCT 116 cells incubated with or without 50 μM 2-BP. Scale bars, 5 μm. Intensity profiles of LAMP1 (green lines) and EGFR (red lines) co-localization signal were shown in plotted lines at three random sites. (D) Representative immunofluorescence images for EGFR (red), LAMP1 (green), and DAPI (blue) of BSA-PA/OA (PA 100 μM, OA 200 μM) treated control (EGFR-WT) and EGFR palmitoylation-deficient (EGFR-9CS) HCT 116 cells. Scale bars, 5 μm. Intensity profiles of LAMP1 (green lines) and EGFR (red lines) co-localization signal were shown in plotted lines at three random sites. (E) Representative immunofluorescence images for EGFR (red), F-actin (green), and DAPI (blue) of BSA-PA/OA (PA 100 μM, OA 200 μM) treated HCT 116 cells incubated with or without 50 μM 2-BP. Scale bars, 10 μm. (F) Representative immunofluorescence images for EGFR (red), F-actin (green), and DAPI (blue) of BSA-PA/OA (PA 100 μM, OA 200 μM) treated control (EGFR-WT) and EGFR palmitoylation-deficient (EGFR-9CS) HCT 116 cells. Scale bars, 10 μm. BSA, bovine serum albumin; PA, palmitic acid; OA, oleic acid; 2-BP, 2-bromopalmitate; CHX, cycloheximide.

Figure S7

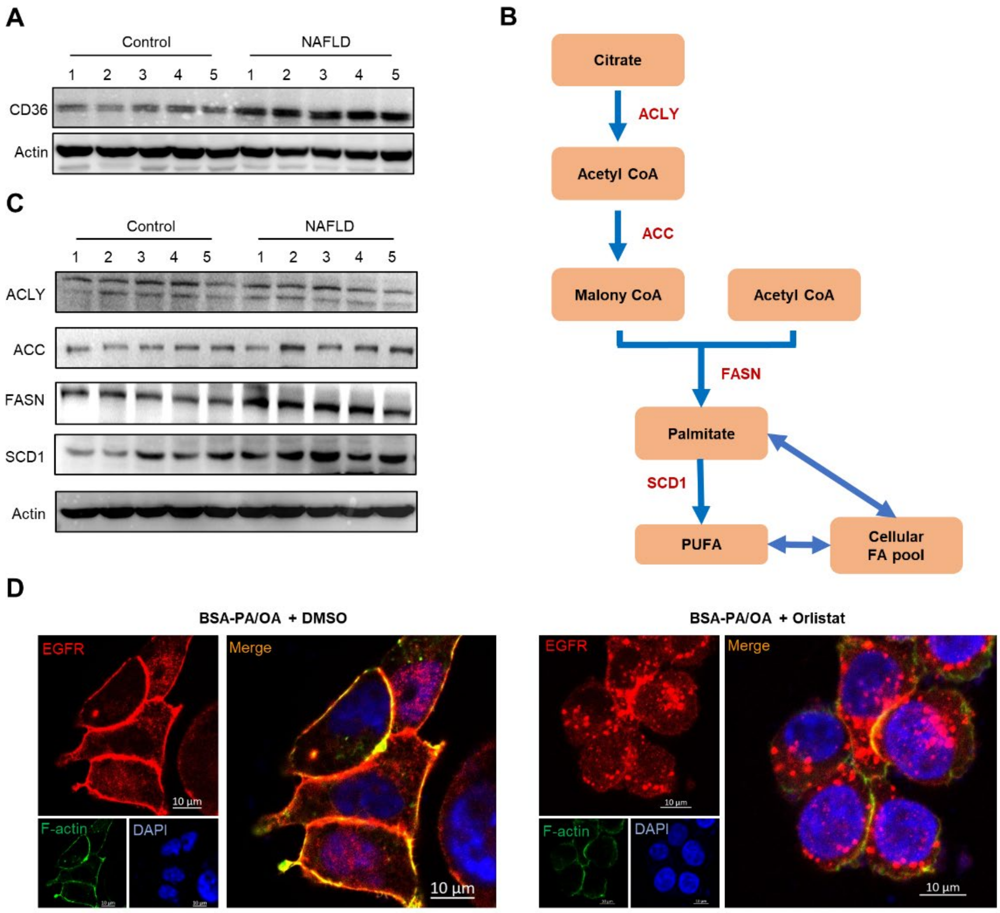

Figure S7 NAFLD metabolic niche activates *de novo* lipogenesis in metastatic CRC cells. (A) The protein level of CD36 of HCT 116 xenografts in control mice and NAFLD mice by western blot assay (n=5). (B) Schematic diagram representing the pathway of *de novo* lipogenesis. (C) The protein level of *de novo* lipogenesis enzymes ACLY, ACC, FASN, SCD1 and of HCT 116 xenografts in control mice and NAFLD mice by western blot assay (n=5). (D) Representative immunofluorescence images for EGFR (red), F-actin (green), and DAPI (blue) of BSA-PA/OA (PA 100 μM, OA 200 μM) treated HCT116 cells incubated with or without 10 μM orlistat. Scale bars, 10 μm. BSA, bovine serum albumin; PA, palmitic acid; OA, oleic acid; ACLY, ATP-citrate

lyase; ACC, Acetyl-CoA Carboxylase; FASN, fatty acid synthase; SCD1, stearoyl-CoA desaturase 1; PUFA, polyunsaturated fatty acid; FA, fatty acid.

Figure S8

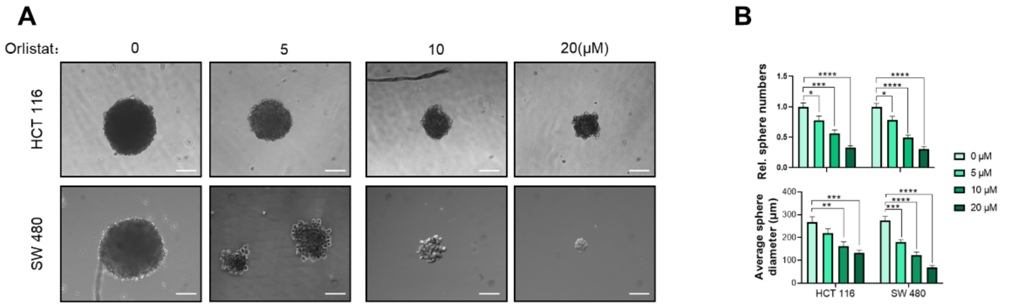

Figure S8 Inhibiting palmitate biosynthesis with orlistat suppresses CRC cell stemness in NAFLD. (A) Sphere formation assay in BSA-PA/OA (PA 100μM, OA 200μM) treated HCT 116 and SW 480 cells incubated with or without orlistat at indicated doses. Representative photos were taken on Day 7 after seeding in first passage, scale bar represents 100 μm. (B) Sphere numbers and diameters were determined and plotted (n=3). Significance was determined by one-way ANOVA, *$P<0.05$, **$p<0.01$, ***$p<0.001$.

Supplementary tables

Table S1. Reagents

**Reagents**	**Source**	**Identifier**
**Antibodies**		
Oct4	CST	2750S, RRID: AB_823583

SOX2	Abcam	ab97959, RRID: AB_2341193
Nanog	Epitomics	3369-1, RRID: AB_10635923
EGFR	Abcam	AB52894, RRID: AB_869579
EGFR	Bimake	A5858
β-Actin	Proteintech	HRP-60008, RRID: AB_2819183
GAPDH	Proteintech	HRP-60004, RRID: AB_2737588
Tubulin	Proteintech	HRP-66031, RRID: AB_2687491
FASN	Selleckchem	A5447
CD36	Affinity	DF13262, RRID: AB_2846281
SCD1	CST	2794, RRID: AB_2183099
LAMP1	Abcam	Ab25630, RRID: AB_470708
ACLY	CST	4332, RRID: AB_2223744
**Reagents and Recombinant Proteins**		
2-BP	Sigma	238422
Palm B	Millipore	3457644
ML348	Targetmol	T3439
NH_4Cl	Aladdin	A116373
Pep A	Targetmol	T3695
Cycloheximide	Sigma	01810
EGF	Novus	NBP2-34952
FGF	Novus	NBP2-34921
LIF	Novus	NBP2-34935

B27	Gibco	17504-049
Orlistat	MCE	HY-B0218/cs-2165
BSA	Biofroxx	EZ2921B398
Palmitate acid	Sigma	P0500
Oleate acid	Sigma	O1008
Oil Red	Sloarbio	G1260
BODIPY	Invitrogen	D3922
MG 132	Targetmol	T2154
RIPA	Beyotime	P0013
DMSO	Solarbio	D8371
TWEEN-20	Solarbio	T8220
Collogenase-I	Biofroxx	EZ4567C110
Collogenase-II	Biofroxx	EZ4567A109
Luciferase potassium salt	Beyotime	St198-500mg
Puromycin	Solarbio	P8230
DAPI	Chemcruz	SC-24941
Lipofectamine 3000	Invitrogen	L3000-008
Blasticidin	Solarbio	B9300
NEM	sigma	E3826
Goat anti-Mouse IgG (H+L) Highly Cross-Adsorbed Secondary	Invitrogen	A32742, RRID: AB_2762825

Antibody, Alexa Fluor™ Plus 594		
Goat anti-Rabbit IgG (H+L) Highly Cross-Adsorbed Secondary Antibody, Alexa Fluor™ Plus 594	Invitrogen	A32740, RRID: AB_2762824
Goat Anti-Rabbit IgG HRP	Affinity	S0001
Goat Anti-Mouse IgG HRP	Affinity	S0002

Table S2. Sequences of oligonucleotide primers for shRNA

**Genes**	**Primer sequence (5’-3’)**
shRNA for EGFR #1	CAGCTATGAGATGGAGGAAGA
shRNA for EGFR #2	CGGCGTCCGCAAGTGTAAGAA

Supplementary methods

Western blotting analysis

HCT 116 and SW480 cells are harvested, washed with PBST, and lysed on ice with

RIPA buffer (Beyotime, China) containing a protease inhibitor cocktail (Roche) for 30 minutes. Total protein was extracted and quantified by a BCA Protein Assay Kit (Beyotime, China) as the manufacturer's instructions suggested. Protein samples were separated by electrophoresis in a 10% gel and then transferred to a PVDF membrane (Millipore). The membrane is blocked and incubated with the primary antibody at 4 °C overnight. On the second day, the membrane was washed 3 times with PBST for 5 min and then incubated with the HRP-linked secondary antibody (cell signaling technique, USA) for 1 h at room temperature. Visualization and detection of band strength with conducted with an enhanced chemiluminescence detection system (Bio-Rad Labs) [1]. Primary antibodies against Oct4 (RRID: AB_823583), Nanog (RRID: AB_10635923), SOX2 (RRID: AB_2341193), EGFR (RRID: AB_869579), GAPDH (RRID: AB_2737588), tubulin (RRID: AB_2687491), and β-actin (RRID: AB_2819183) were obtained from manufacturers listed in Table S1.

Sphere formation assay

Spheroids were cultured in ultra-low compliance (ULA) polystyrene plates (Corning), incubated with DMEM/F12 added with 20 mg/ml insulin (sigma), 20 ng/ml EGF (R&D system), 10 ng/ml bFGF (R&D system), 3 mg/ml D-glucose (sigma) and 1% penicillin-streptomycin (pan-biotech). Briefly, for sphere formation assays, 1, 000 live single cells were inoculated in 2 ml medium per well in 6-well ULA plates. After 7 days in 5% CO_2 at 37 °C, take a microscopic photograph of the sphere. Then the first passage spheroids were trypsinized and plated in 6-well plates at a density of 1, 000 cell/well in the media

described above. After 10 days in 5% CO_2 at 37 °C, take a microscopic photograph of the sphere. The number and size of colonies were analyzed using ImageJ (RRID:SCR_003070) [2].

Immunohistochemistry assay

Prepare and preserve tissue samples by paraffin embedding and mount them on 3-aminopropyltrioxysilane-coated slides, dewax and block in hydrogen peroxide/methanol solution. Perform antigen retrieval by pressure cooking of samples in 0.08% citrate buffer for 20 minutes. Before used for tissue sectioning, the antibody is titrated with normal control tissue to determine the dilution with optimal sensitivity and specificity. Staining results are visualized by sequential incubation with components of the Vision+ assay system (EnVision + /HRP/Mo, Dako, Glostrup, DK). Paraffin-embedded liver sections were stained with H&E and stained using Oil Red O and BODIPY staining to visualize lipid accumulation patterns in frozen liver sections [1].

ShRNA transfections

The ShRNA oligonucleotides used were ShEGFR (2 targets), constructed by GENECHEM Biotech at Shanghai, China. ShRNA (5 ng) was transfected into cells using Lipofectamine 3000 (Thermo Fischer Scientific) transfection reagent in accordance with the supplier's recommendations.

Plasmids

Plasmids for wild type EGFR(WT) and the palmitoylation mutant EGFR(9CS) plasmid (C775S, C781S, C797S, C818S, C939S, C950S, C1049S, C1058S, and C1146S) was designed and constructed by Hanbio Biotechnology (Shanghai, China), the vector of which was pcDNA3.1, and the plasmid have been verified by DNA sequencing.

Supplementary References

1 Ou J, Peng Y, Yang W, Zhang Y, Hao J, Li F, et al. ABHD5 blunts the sensitivity of colorectal cancer to fluorouracil via promoting autophagic uracil yield. Nat Commun. 2019;10:1078.

2 Gu Y, Chen Y, Wei L, Wu S, Shen K, Liu C, et al. ABHD5 inhibits YAP-induced c-Met overexpression and colon cancer cell stemness via suppressing YAP methylation. Nat Commun. 2021;12:6711.
