# Supplementary material for: Non-alcoholic fatty liver disease promotes liver metastasis of colorectal cancer via fatty acid synthase dependent EGFR palmitoylation
Source: Cell Death Discov. 2024 Jan 23;10:41. doi: 10.1038/s41420-023-01770-x (PMC10805926; doi:10.1038/s41420-023-01770-x)

Figure 2

Full unedited gel for Figure 2D.

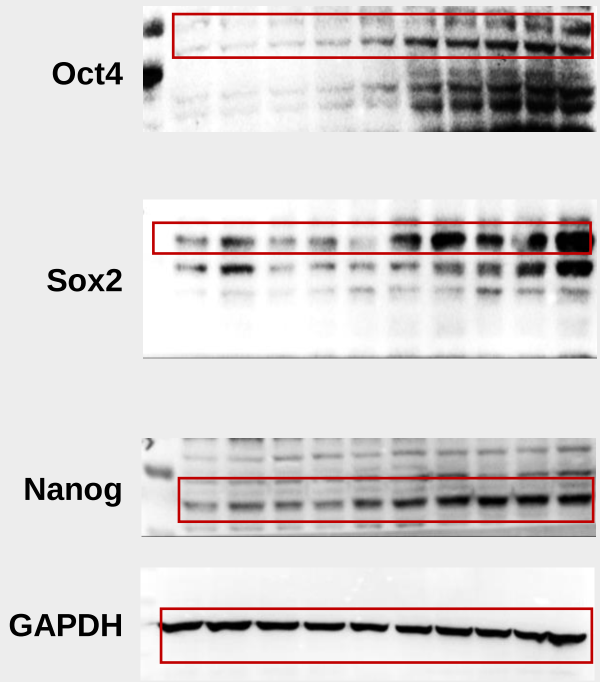

Full unedited gel for Figure 2F left.

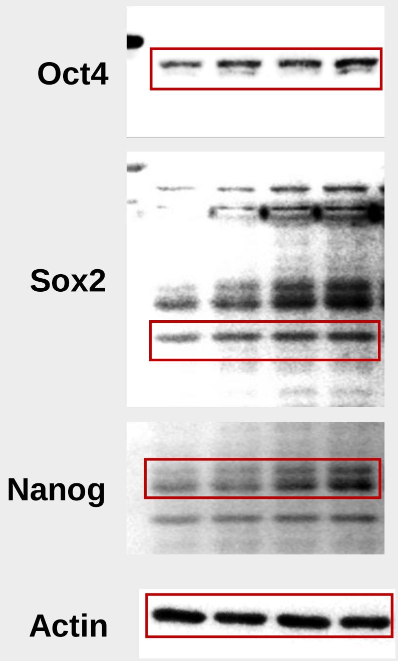

Full unedited gel for Figure 2F Right.

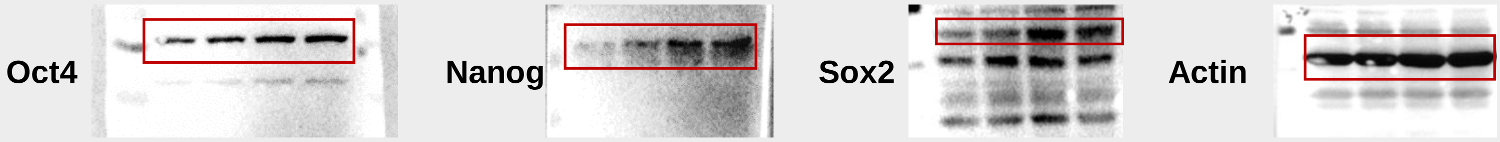

Figure 3

Full unedited gel for Figure 3F.

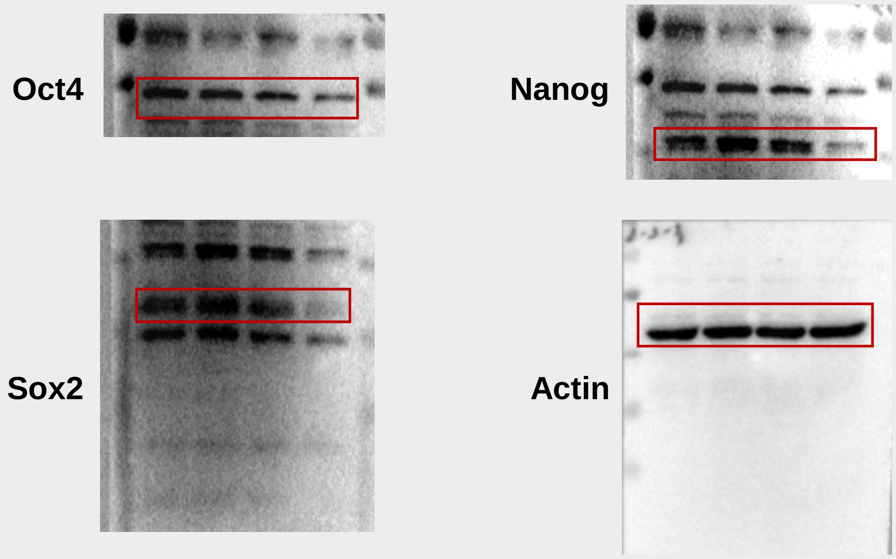

Full unedited gel for Figure 3H left.

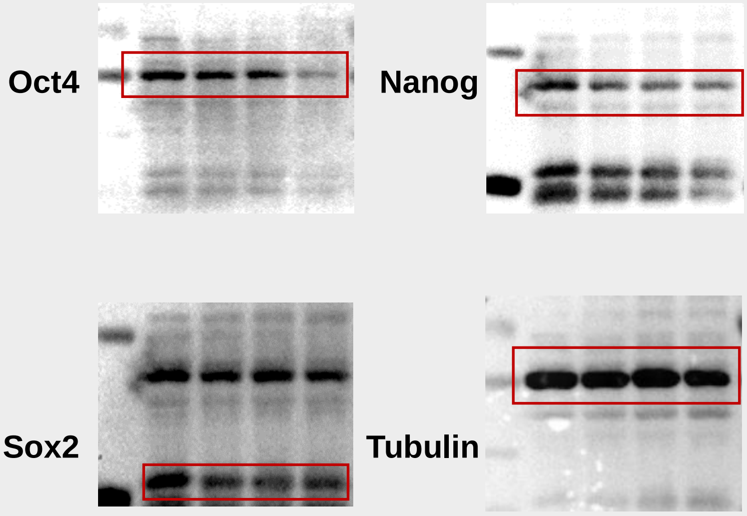

Full unedited gel for Figure 3H Right.

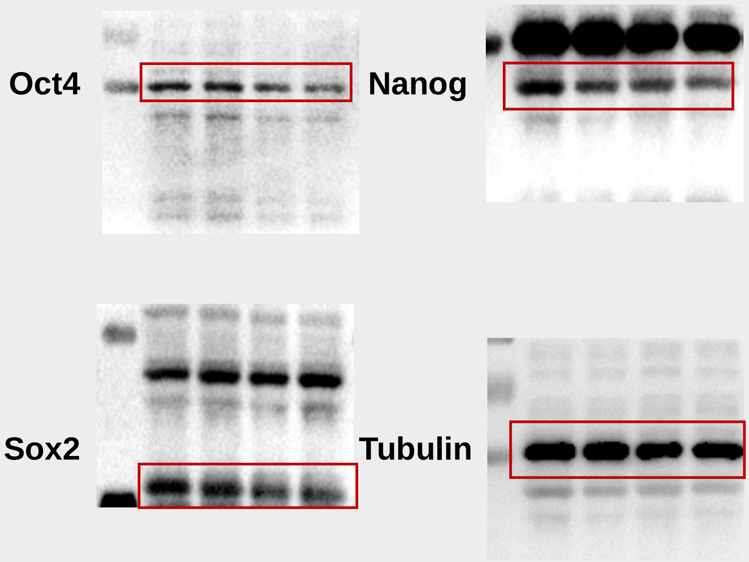

Figure 4

Full unedited gel for Figure 4B.

EGFR

Tubulin

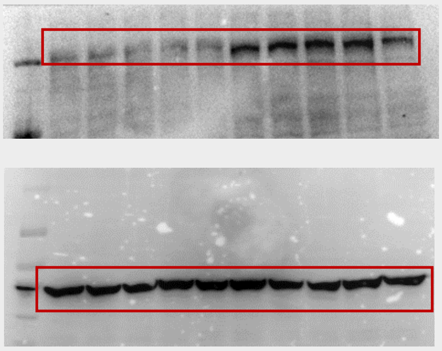

Full unedited gel for Figure 4C.

EGFR

GAPDH

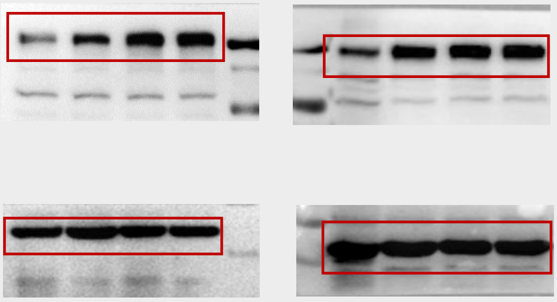

Full unedited gel for Figure 4D.

EGFR

GAPDH

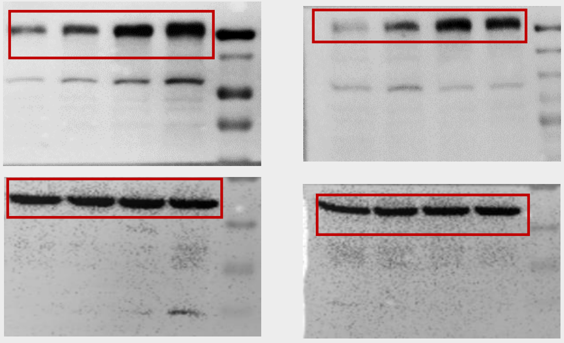

Full unedited gel for Figure 4F.

Palm-
EGFR

EGFR

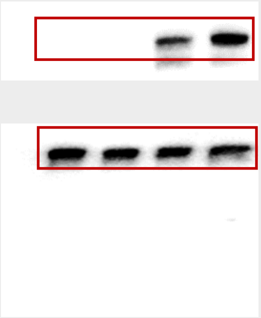

Full unedited gel for Figure 4E.

Palm-
EGFR

EGFR

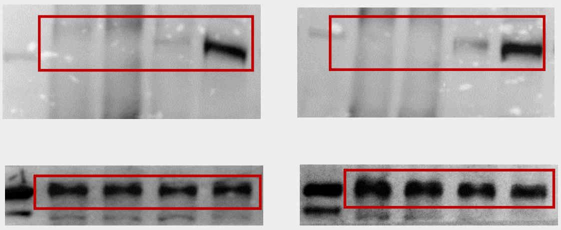

Full unedited gel for Figure 4G.

Palm-
EGFR

EGFR

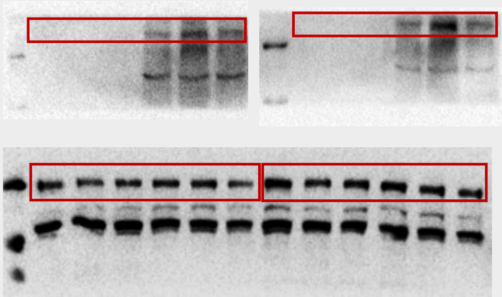

Full unedited gel for Figure 4H.

EGFR

GAPDH

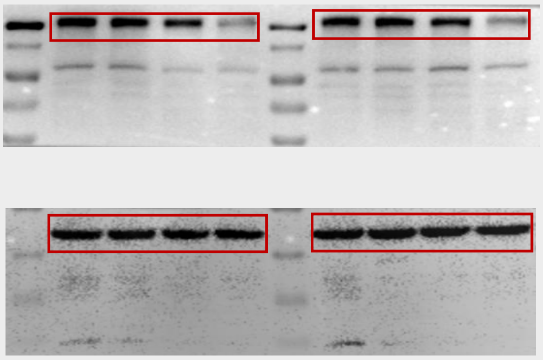

Full unedited gel for Figure 4I.

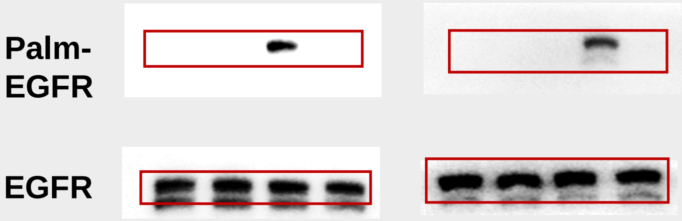

Full unedited gel for Figure 4J.

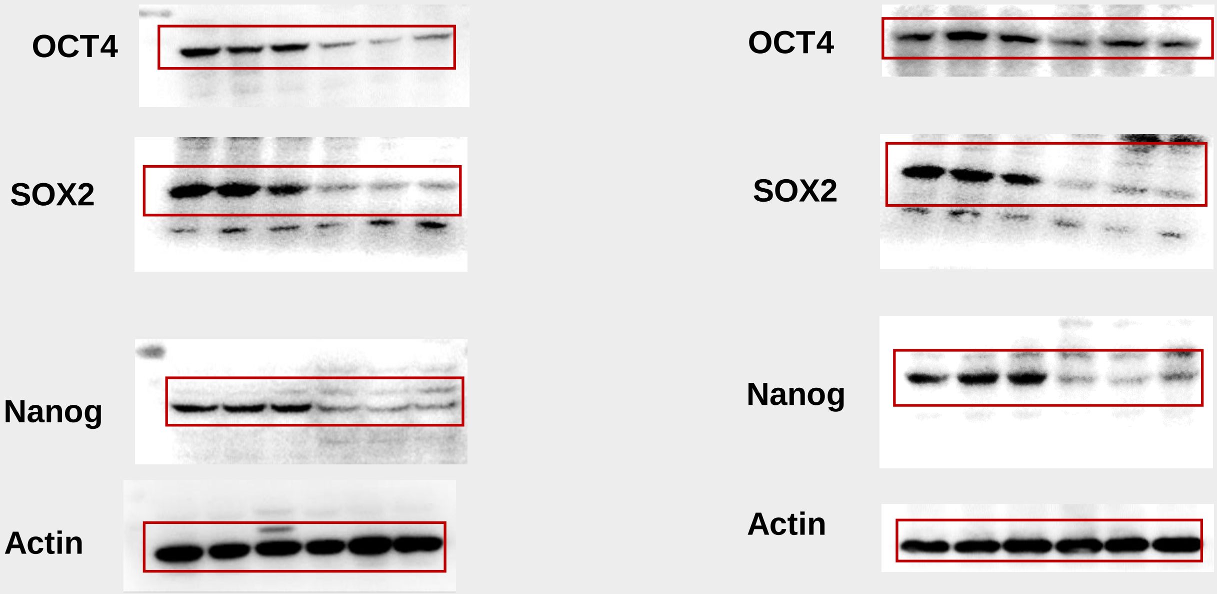

Figure 5

Full unedited gel for Figure 5A.

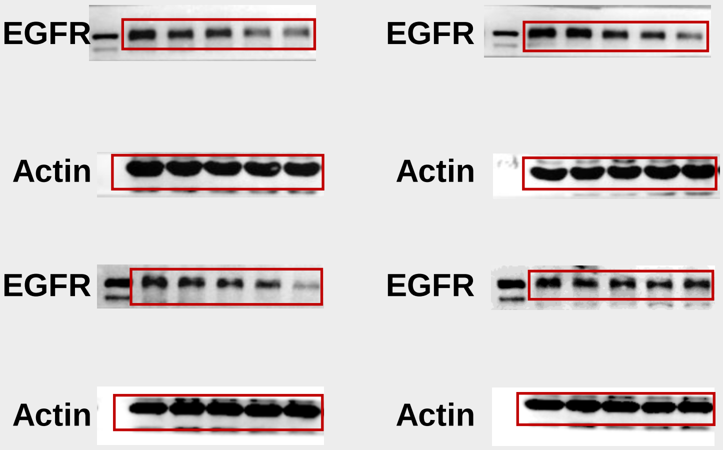

Full unedited gel for Figure 5B.

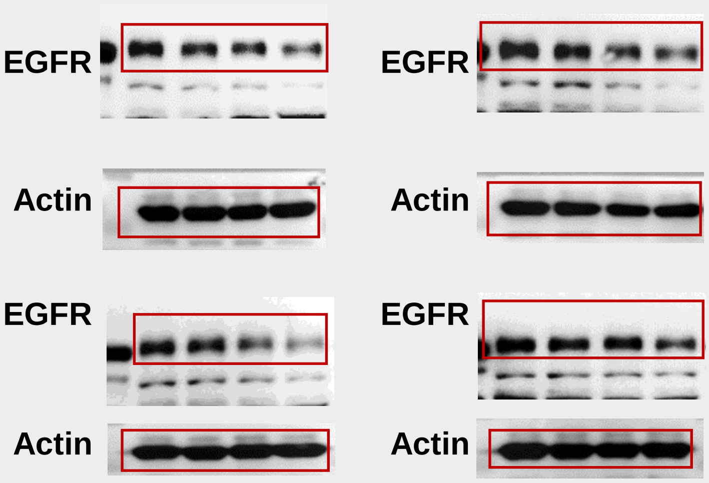

Full unedited gel for Figure 5C.

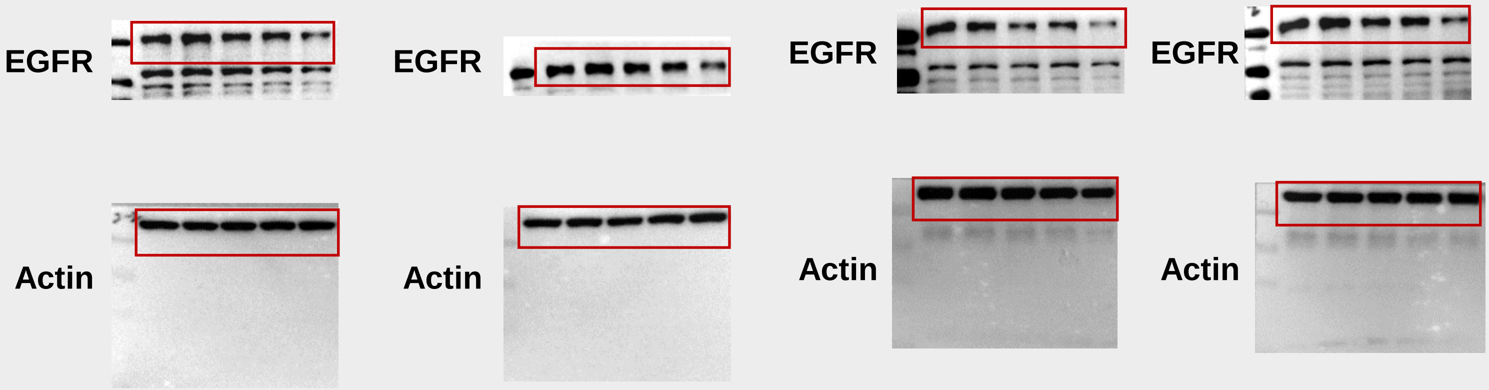

Full unedited gel for Figure 5D.

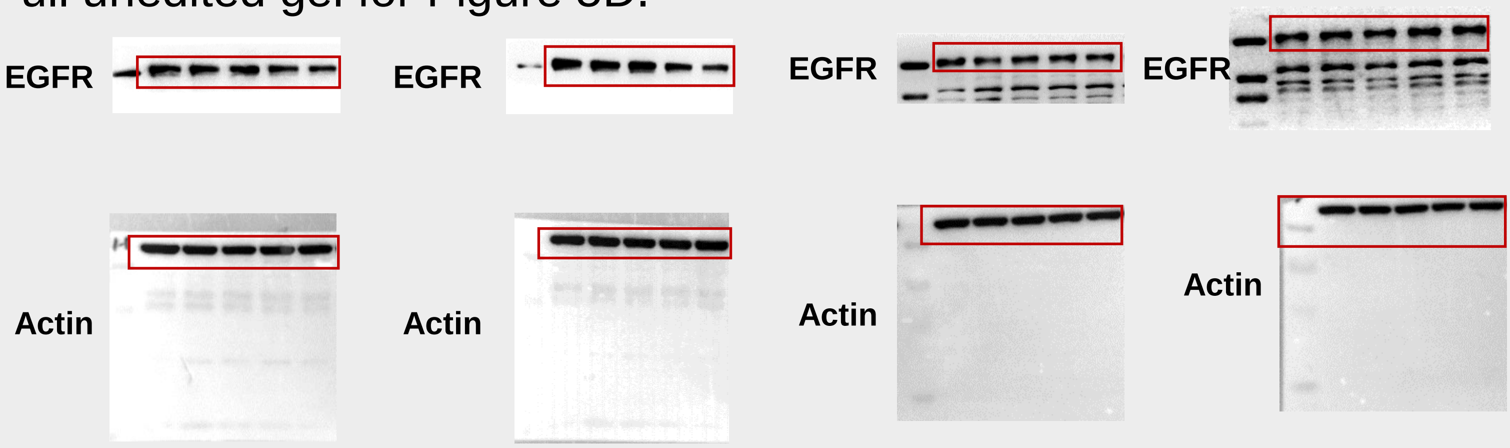

Figure 6

Full unedited gel for Figure 6B.

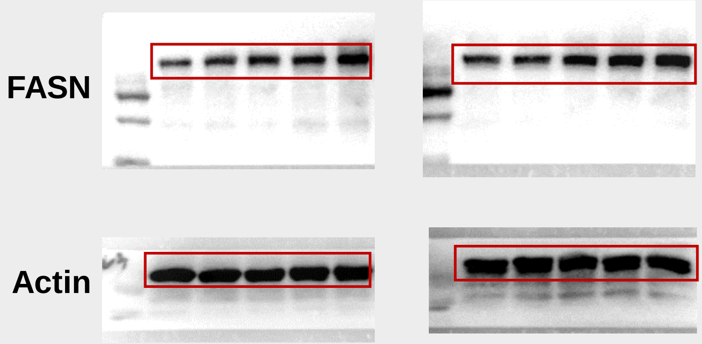

Full unedited gel for Figure 6E.

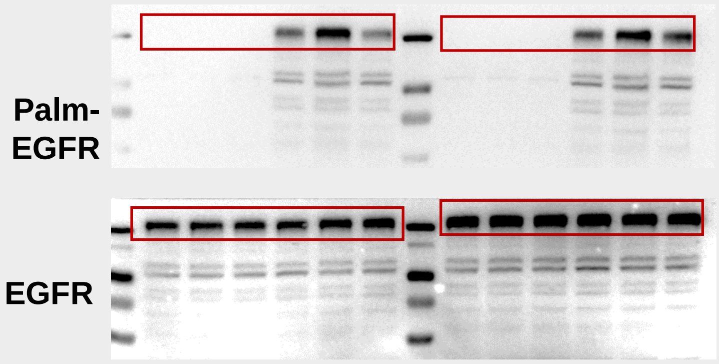

Full unedited gel for Figure 6F.

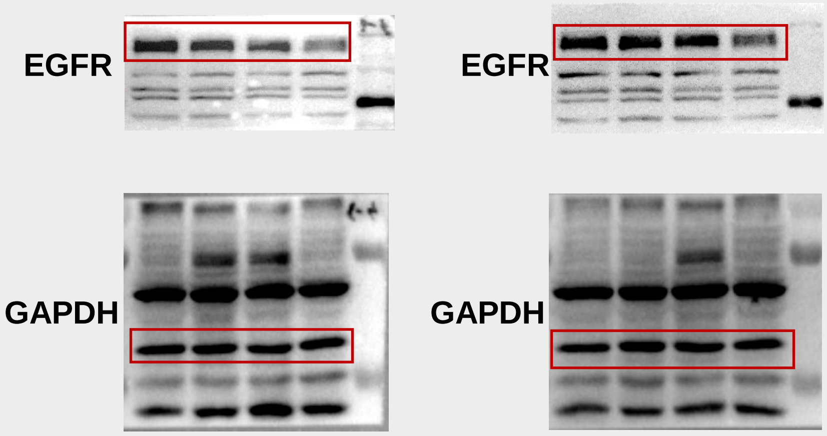

Full unedited gel for Figure 6H.

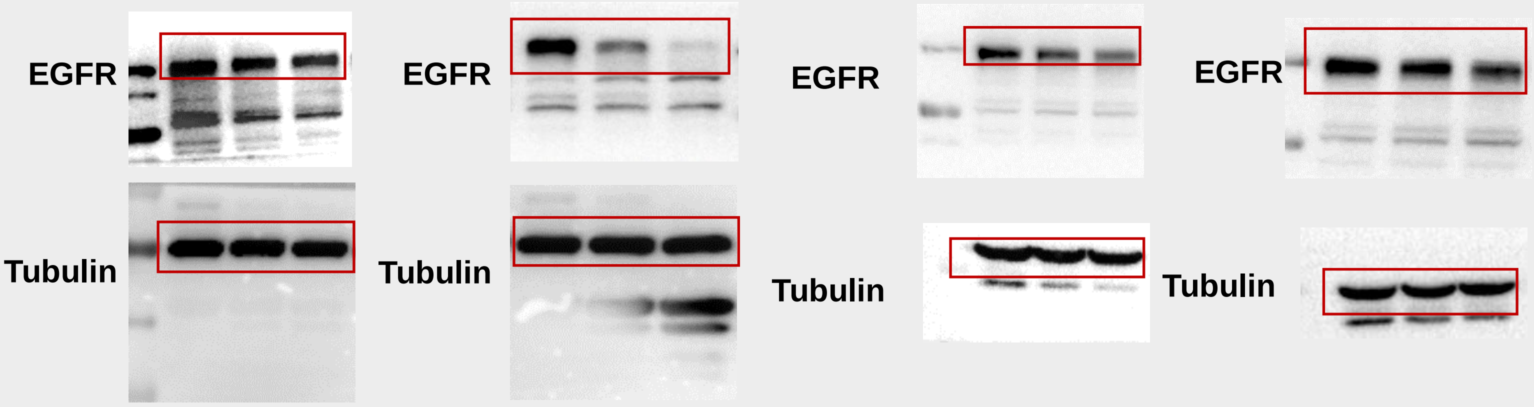

Full unedited gel for Figure 6I.

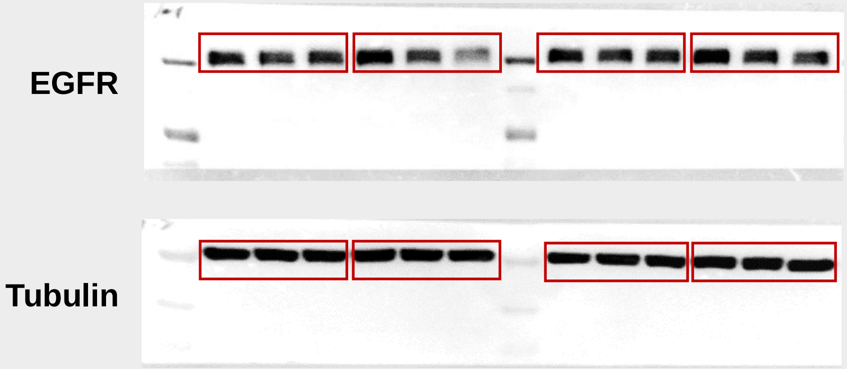

Figure 7

Full unedited gel for Figure 7B.

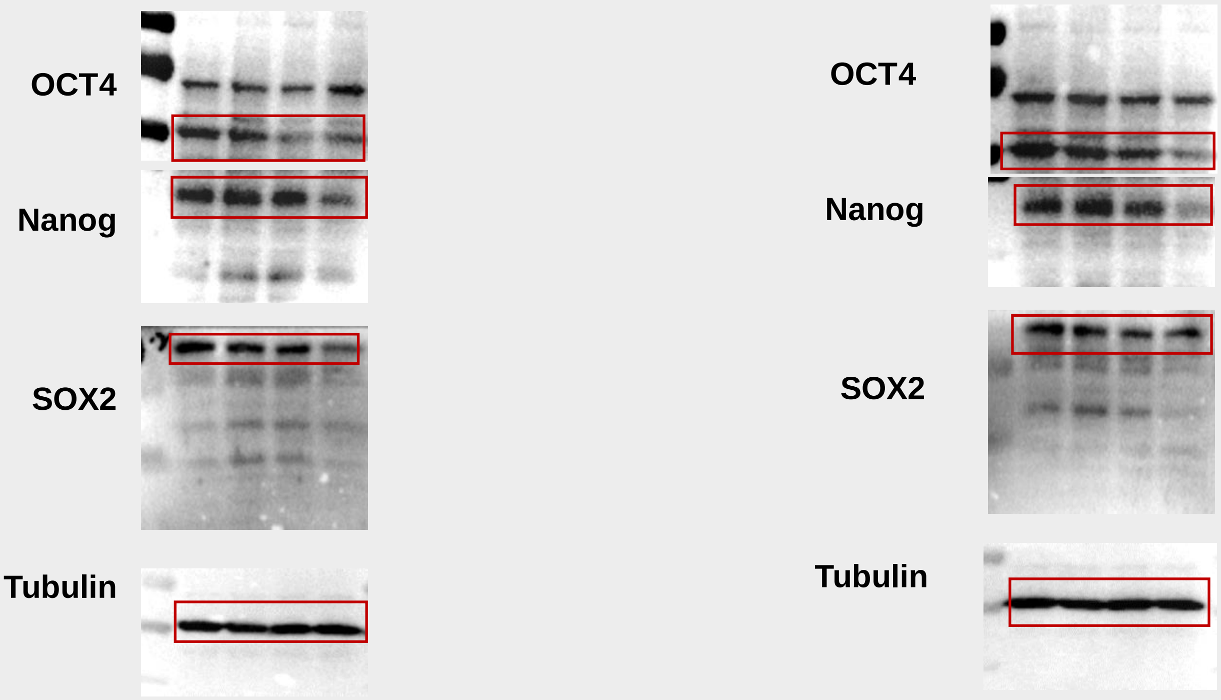

Supplement: Supplementary file 2 — Original Data File [file 41420_2023_1770_MOESM2_ESM.pdf]
